# The GacS/GacA two-component system strongly regulates antimicrobial competition mechanisms of *Pseudomonas fluorescens* MFE01 strain

**DOI:** 10.1128/jb.00388-24

**Published:** 2025-01-23

**Authors:** Charly A. Dupont, Yvann Bourigault, Héloïse Biziere-Maco, Amine M. Boukerb, Xavier Latour, Corinne Barbey, Julien Verdon, Annabelle Merieau

**Affiliations:** 1 Laboratoire de Communication Bactérienne et Stratégies Anti-infectieuses (CBSA UR4312, formerly LMSM EA4312), Univ Rouen Normandie, Université Caen Normandie, Normandie Univ, Rouen, France; 2 International Research Federation NOR-SEVE, University of Sherbrooke 7321 , Sherbrooke, Québec, Canada; 3 Normandie University, Rouen, France; 4 Biocontrol and Biostimulation for Agroecology Association (ABBA), Paris, France; 5 Laboratoire Ecologie & Biologie des Interactions, UMR CNRS 7267, Université de Poitiers 27077 , Poitiers, Nouvelle-Aquitaine, France; University of California San Francisco, San Francisco, California, USA

**Keywords:** GacA/GacS, Volatile compounds, Type 6 Secretion System, *Pseudomonas fluorescens*

## Abstract

*Pseudomonas fluorescens* MFE01 is an environmental bacterium characterized by an hyperactive type 6 secretion system (T6SS) and a strong emission of volatile organic compounds (VOCs). In a previous study, a transposition mutant, 3H5, exhibited an inactive T6SS and altered VOC emission. In 3H5, the interruption of *trpE* gene by the transposon was insufficient to explain these phenotypes. To determine the actual impact of this insertion, a comparative transcriptomic analysis was performed on the two-component system GacS/GacA, known to influence numerous phenotypes in *Pseudomonas*. The results demonstrated that the *gacS* gene is less expressed in 3H5 than in MFE01. Phenotypic analysis of a *gacS* deletion mutant, Δ*gacS*, confirmed many similarities between Δ*gacS* and 3H5. Indeed, Δ*gacS* exhibited an inactive T6SS and an altered VOC emission profile. In-depth analysis of volatilomes and phenotypes correlated with the decrease in *gacS* transcription, highlighting that the emission of 1-undecene is under the strict control of GacS/GacA. This study confirms that 1-undecene is not the sole volatile molecule responsible for MFE01’s inhibition of *Legionella*. Moreover, MFE01 has antimicrobial activity against the phytopathogenic oomycetes *Phytophthora infestans* via hydrogen cyanide (HCN) emission, which is also controlled by GacS. In MFE01, GacS/GacA is also a partial positive regulator of other VOC emission, whose reduced emission in 3H5 coincides with the decrease in *gacS* transcription.

**IMPORTANCE:**

Our model strain *Pseudomonas fluorescens* MFE01 uses an active type VI secretion system (T6SS) and volatile compounds (VCs) to outcompete other microorganisms in the natural environment. By investigating the cellular mechanism regulating the production of these weapons, we identified the two-component system GacS/GacA. Indeed, GacS cellular membrane sensor plays a crucial role in regulating T6SS activity and VC emission. Among the latter, 1-undecene and hydrogen cyanide are strong aerial inhibitors of the *Legionella* human pathogen and the *Phytophtora infestans* major plant pest, respectively. The aim is to improve the understanding of the regulation of these volatile molecule emission and the critical role of a global regulator in both plant and human health.

## INTRODUCTION

In order to adapt to various environmental stimuli, bacteria have developed a large number of transmembrane sensors, including two-component system (TCS) sensors. Sensors of these TCS are autophosphorylated upon detection of specific signals, and then the perception of this signal is transduced by phosphorylating cytoplasmic transcription regulators. This modification enables regulators to bind DNA and modulate the transcription of specific genes, thereby facilitating bacterial adaptation to the encountered signal (1–3). In *Pseudomonas* species, the GacS/GacA TCS is highly conserved. GacS, the histidine kinase sensor component, autophosphorylates upon detecting an as-yet-unknown ligand, and transfers the phosphate through a phospho-relay to GacA, the transcriptional regulator (2, 4). Modulation of GacA phosphorylation occurs through two connectors, RetS and LadS, with negative or positive effects, respectively (4). Phosphorylated GacA binds to target gene promoters, inducing the expression of small non-coding RNAs (ncRNA: RsmX, RsmY, and RsmZ), which sequester translation-inhibiting proteins of the RsmA family acting by binding to mRNAs (RsmA, RsmE, and RsmI, and functional homologs of CsrA in *Esherichia coli*) (4). The Gac/Rsm pathway regulates virulence, mobility, metabolic transitions, biofilm formation, secretion systems, emission of volatile organic compounds (VOCs), and bacterial communication in a variable way, depending on the species or strain (5–7). As an example of this major regulatory ability, the insertion of a transposon into the *gacS* gene of *Pseudomonas fluorescens* SBW25 modifies the expression of more than 700 genes, thus impacting social phenotypes, plant growth promotion activity, and microbial competition (8). Particularly, this Gac/Rsm is known for regulating the synthesis of hydrogen cyanide (HCN) (9, 10). Some *Pseudomonas* synthesize this inorganic volatile compound by oxidative decarboxylation of glycine via the HCN synthase, which is coded by the *hcnABC* operon (11–13). HCN can inhibit cellular respiration by complexing with the metal ions that make up the prosthetic groups of cytochrome c oxidase (complex IV in the respiratory chain), instantly preventing any production of ATP. Accordingly, HCN promotes competitive fitness of HCN emitters against several fungi and prokaryotes, including in the context of hosting provided by mammals, plants, insects or nematodes (13–16).

*Pseudomonas fluorescens* MFE01 strain inhibits some human and plant pathogens via the emission of volatile organic compounds (VOCs) or its type 6 secretion system (T6SS) (17, 18). MFE01 is indeed characterized by its high emission level of 1-undecene and by an offensive and hyperactive T6SS. VOCs are small, gaseous molecules (MW <500 Da) under environmental conditions due to their lipophilic part, low boiling point, and high vapor pressure (19). VOCs are notably described to be involved in interactions between bacteria, plants, and fungi (19). T6SSs are contractile protein nanomachines that translocate effectors, predominantly toxins, into nearby targets or in the extracellular environment (20–23). The contraction of the T6SS sheath (TssB and TssC proteins) propels needle-containing Hcp proteins and effectors outside of the cell. T6SSs are involved in virulence, bacterial competition, and communication (24). Therefore, VOCs and T6SSs seem to be intraspecific communication signals in the MFE01 strain, which does not secrete acyl homoserine lactone or alkyl quinolone, quorum sensing autoinducers used by several *Pseudomonas* species (24, 25). A previous study highlighted a possible correlation between the emission of 1-undecene and the activity of T6SS in MFE01 (17). Indeed, a MFE01 transposition mutant named 3H5 with an insertion of the transposon into the *trpE* gene (involved in tryptophan metabolism) that emitted a low level of 1-undecene had lost its anti-*Legionella* activity and had an inactive T6SS. A reversion mutant was obtained by chromosomal reintroduction of *trpE* at the transposon site. This strain (3H5-rev) showed a total restoration of the anti-*Legionella* activity, T6SS, and VOC emission profile. Short-read mapping did not reveal any single nucleotide polymorphism (SNP) between the wild-type MFE01 and 3H5. In addition, an in-frame *trpE* mutant showed no alteration of these phenotypes. This previous study led us to conclude that all phenotypes of 3H5 resulted from unexplained polar effects induced by the Tn5 transposon insertion into *trpE*. This work ruled out the possibility of T6SS regulation and 1-undecene emission by tryptophan anabolism or catabolism. Since then, a complementary study has rejected the hypothesis of T6SS regulation by 1-undecene but without explaining the previously observed phenotypes in 3H5 (25). The present study does not aim to understand how the insertion of the transposon could have generated polar effects but rather to find the smallest common denominator between the phenotypes affected in 3H5. The phenotypes affected in the 3H5 mutant have been described to be regulated by the Gac/Rsm pathway in other strains, leading us to hypothesize a perturbation of this pathway in the 3H5 mutant (6, 26). Here, in order to explore this hypothesis, we first characterized the Gac/Rsm system of MFE01 *in silico* and carried out quantitative RT-PCR experiments targeting genes of the Gac/Rsm pathway in 3H5, *trpE* mutant and MFE01 strains. We then constructed an in-frame *gacS* deletion mutant and compared its competitive ability against two *Solanum tuberosum* phytopathogens*, Pectobacterium atrosepticum*, and *Phytophtora infestans*, as well as against the human pathogen *Legionella pneumophila*, whose growth is inhibited by the wild-type MFE01 strain, via T6SS or volatile compounds. We demonstrated that the Gac/Rsm system exerts a strict positive regulation on T6SS, 1-undecene and HCN emission in MFE01, which are crucial for its competitive antagonism. Additionally, correlations between *gacS* transcription and the emission of other VOCs suggest new areas for controlling *gacS* expression.

## RESULTS

### *In silico* characterization predicts a complete Gac/Rsm pathway in MFE01

To determine whether the elements of the Gac/Rsm system are present in MFE01, several tblastn searches (https://blast.ncbi.nlm.nih.gov/Blast.cgi) were conducted using protein sequences from the Gac/Rsm pathway elements of various *Pseudomonas* species, in which the Gac/Rsm system has been previously characterized. A schematic representation outlines this signaling pathway’s operation (Fig. S1). The sequences were obtained from the *Pseudomonas* Genome database (https://www.pseudomonas.com/). For the ncRNAs, blastn was used with the sequences of the referenced genes for RsmX, RsmY, and RsmZ. The reference genomes of *Pseudomonas aeruginosa* PAO1, *P. fluorescens* SBW25, *Pseudomonas ogarae* F113 (formerly *Pseudomonas fluorescens* F113), *Pseudomonas protegens* Pf-5, and *Pseudomonas protegens* CHA0 were used. This *in silico* analysis showed the presence of genes encoding the proteins GacS (accession number: PP328558), GacA (accession number: PP328559), LadS (accession number: PP328560), RetS (accession number: PP328561), three proteins belonging to the carbon storage regulator (Csr) family, here named RsmE (accession number: PP328562), RsmI (accession number: PP328563), and RsmA (accession number: PP328564), and three small non-coding RNAs RsmX (accession number: PP328567), RsmY (accession number: PP328566), and RsmZ (accession number: PP328565) (Table 1). The highest identity percentages were observed with the Gac/Rsm system elements of *P. ogarae* F113 (Table 1). The sequence alignments for these different elements were performed using the MUSCLE tool implemented within the seaview5 software (https://doua.prabi.fr/software/seaview) (27) and are shown in supplementary figures (Fig. S2 to S7).

**TABLE 1:** Components of the Gac/Rsm system present in *P. fluorescens* MFE01^*a*^

MFE01	PA01		SBW25		F113		Pf-5		CHA0	
	% identity	% query cover	% identity	% query cover	% identity	% query cover	% identity	% query cover	% identity	% query cover
GacS	70.5	98	85.7	100	91.7	100	87.7	100	87.7	100
GacA	86.9	100	96.2	100	99.1	100	96.7	99	96.7	99
LadS	63.8	96	72.2	96	82.1	98	74.4	98	74.4	98
RetS	60.3	99	34.1	54	85.7	99	85.4	100	85.6	100
RsmE	67.2 RsmA	100	96.9 CsrA1	100	100 RsmE	100	95.3 RsmE	100	95.3 CsrA1	100
RsmI	55.6 RsmA	88	65.5 CsrA3	95	70.7 RsmI	95	59.6 RsmE	85	59.6 CsrA1	85
RsmA	80.6 RsmA	100	100.000 CsrA2	98	98.4 RsmA	100	100 RsmA	100	100 CsrA2	100
RsmX	/	/	/	/	87 RsmX	100	88 RsmX	79	87	79
RsmY	72 RsmY	98	97.5	100	94 RsmY	100	95 RsmY	100	95	100
RsmZ	86 RsmZ	51	82.8	99	86.5 RsmZ	99	88.2 RsmZ	97	88	99

^*a*^ /, no significant result.

### Expression of Gac/Rsm pathway genes is decreased in the 3H5 mutant

**Fig 1:**
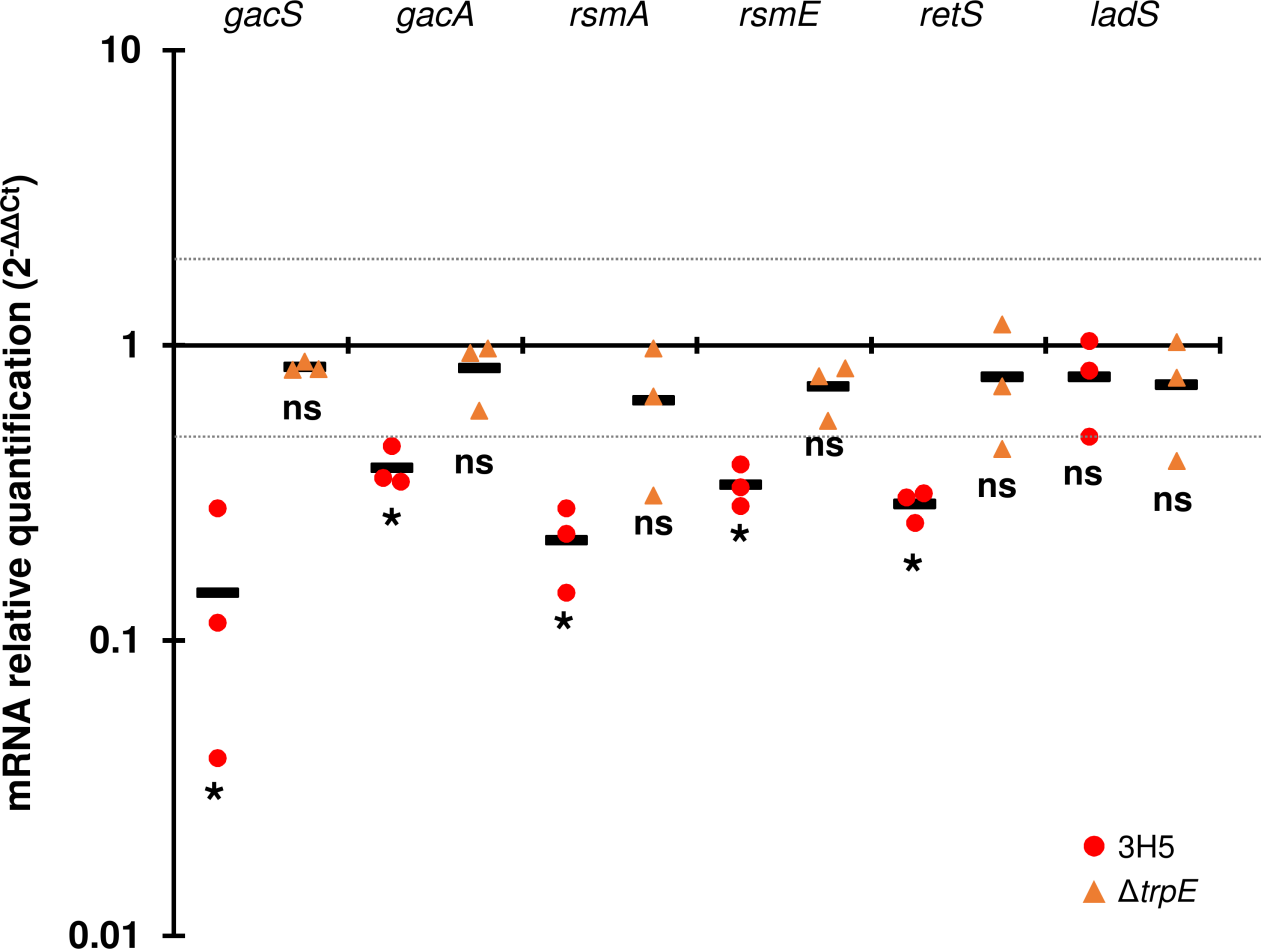
Relative expression of the Gac/Rsm pathway genes in 3H5. The quantity of mRNA from genes in the Gac/Rsm pathway measured in the 3H5 mutant or Δ*trpE* mutant is relative to that obtained in the MFE01 WT strain. Relative expression levels are based on the comparative cycle threshold (Ct) method. Each point represents an independent measurement (*n* = 3). The name of each targeted gene (i.e., *gacS, gacA, rsmA, rsmE, retS*, and *ladS*) is indicated at the top of the graph. Dotted lines represent threshold values for a twofold increase or decrease in relative expression. The expression level of each gene was compared with that of the MFE01 WT strain using a non-parametric Wilcoxon test. A *P*-value < 0.05 indicates statistically significant differences. *, *P* < 0.05; ns, *P* > 0.05.

The transcription level of some Gac/Rsm pathway genes was quantified in both MFE01 wild-type strain (WT), the 3H5 transposition mutant and in-frame *trpE* mutant (Δ*trpE*). The relative expression of each gene in 3H5 and Δ*trpE* was determined by normalizing mRNA quantification to that of the MFE01 WT strain using the *recA* reference gene (Fig. 1). Only results showing a twofold decrease or increase (i.e., relative expressions below 0.5 or above 2) were considered relevant. Whereas the *ladS* mRNA level remained unchanged, the mRNA levels of *gacS*, *gacA*, *retS, rsmA*, and *rsmE* were 6.9, 2.6, 3.4, 4.5, and 3 times lower, respectively, in the 3H5 mutant compared with the WT strain (Fig. 1). No significant changes were observed in the relative expression of these genes in the Δt*rpE* mutant (Fig. 1).

### Deletion of *gacS* inactivates the hyperactive T6SS of MFE01

**Fig 2:**
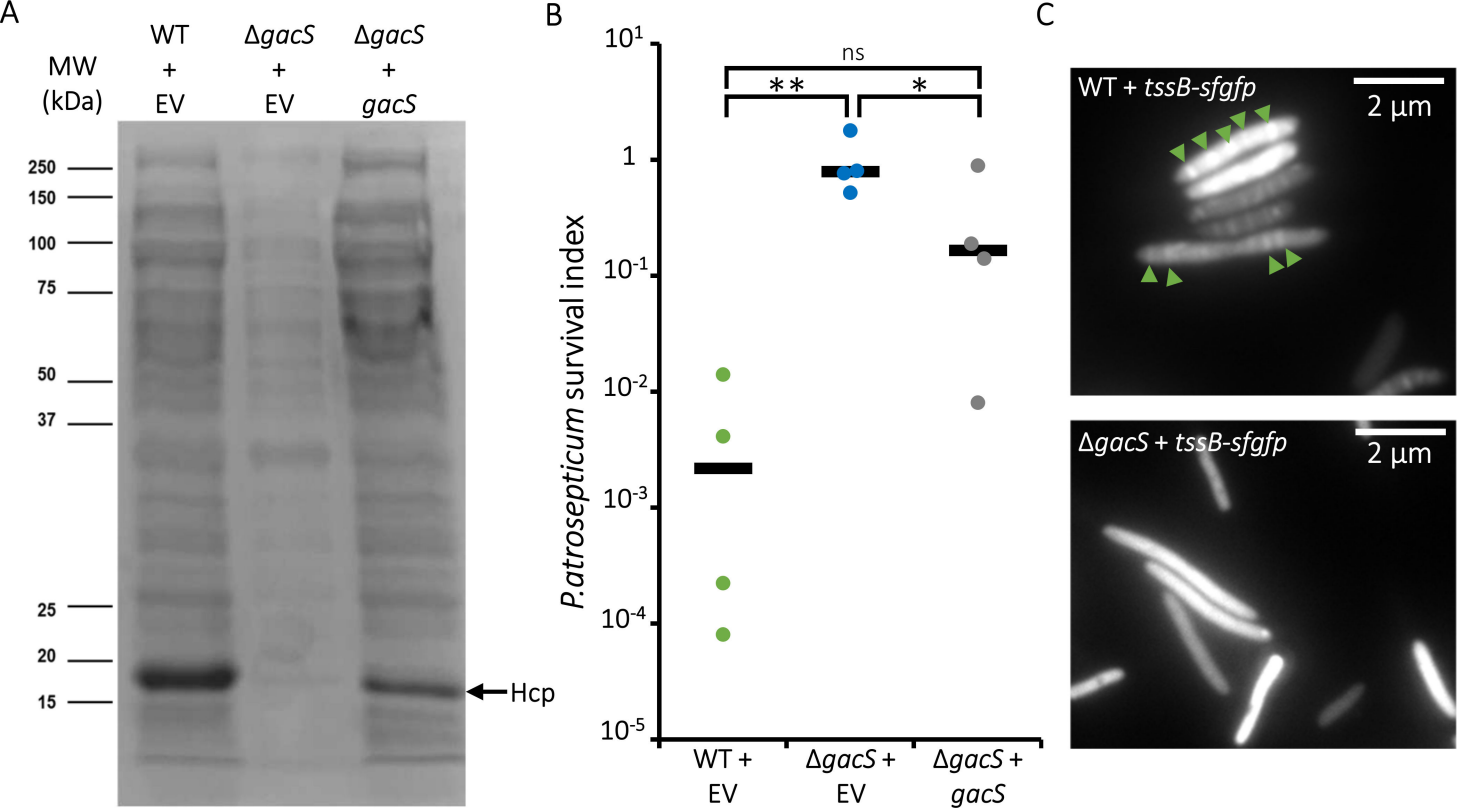
Regulation of MFE01’s T6SS by the Gac/Rsm system. (**A**) Analysis of proteins secreted by *P. fluorescens* MFE01 or its mutants on a 12% acrylamide gel under denaturing conditions (SDS-PAGE). The equivalent of proteins secreted by 2 mL of culture adjusted to OD_580_ = 1 is loaded onto each gel. Hcp is a component of T6SS secreted during T6SS activity, identified by mass spectrometry. (**B**) Killing activity against *Pectobacterium atrosepticum*. Surviving *P. atrosepticum* cells were counted after 4 h of contact with *P. fluorescens* MFE01 or its mutants. The number of surviving *P. atrosepticum* cells in each condition was divided by the number of *P. atrosepticum* cells obtained in the absence of competitor to calculate a survival index. Each point corresponds to an independent sample. The black bar represents the mean. (**C**) Visualization by fluorescence microscopy of MFE01’s T6SS. The WT strain (top image) and the Δ*gacS* mutant (bottom image) were transformed with the plasmid pJN105 containing the translational fusion *tssB-sfGFP*, allowing visualization of the T6SS sheath. Green arrows indicate T6SS sheath. WT+EV : wild-type MFE01 with empty plasmid pJN105. Δ*gacS*+EV: *gacS* mutant with empty plasmid pJN105. Δ*gacS+gacS: gacS* gene mutant with plasmid pJN105 containing the wild-type copy of the *gacS* gene. WT+*tssB-sfgfp*: wild-type *P. fluorescens* MFE01 strain with translational fusion between *tssB* and *sfgfp*. Δ*gacS+tssB-sfgfp: gacS* mutant with translational fusion beteween *tssB* and *sfgfp*. For each experiment, the medium was supplemented with 0.02% arabinose (w/v). Each experiment was repeated at least four times (*n* ≥ 4). Quantitative data were compared via an ANOVA test. A *P*-value < 0.05 indicates significative differences. ns, non-significant; *P < 0.05; **P < 0.01.

To confirm that the phenotypes observed in 3H5 are indeed due to an impact on the Gac/Rsm pathway, an in-frame deletion mutant of the *gacS* gene was constructed. The *gacS* mutant has a slightly higher growth rate than the wildtype (Fig. S8). According to literature, the transcription of the small ncRNAs *rsm*X, Y, and Z genes cannot be activated in a Δ*gacS* mutant (4). This mutant mimics a Gac/Rsm system where GacS does not interact with its ligand, thereby locking the binding of RsmA, RsmE, and RsmI to their target mRNAs. The T6SS activity of this mutant was then analyzed using three different approaches (Fig. 2A, B and C). First, the secretion of Hcp proteins in the culture supernatant (Fig. 2A), a marker of an active T6SS, was performed by protein electrophoresis on acrylamide gel and Coomassie blue staining. A characteristic band was visible at approximately 19 kDa for the wild-type strain containing the inducible arabinose expression empty vector pJN105 (WT + EV) (Fig. 2A). In the Δ*gacS* mutant containing the same empty vector (Δ*gacS* +EV), no Hcp secretion was detectable. Expression of the wild-type *gacS* gene cloned into the plasmid pJN105 partially restored the Hcp protein secretion in the Δ*gacS* mutant (Δ*gacS+gacS*), confirming the essential role of GacS in the activity of this T6SS (Fig. 2A). The overall quantity of proteins secreted by the Δ*gacS*+EV strain, per equal cell quantity, was lower compared with the WT + EV strain. The Δ*gacS+gacS* strain secreted more Hcp proteins than the Δ*gacS*+EV strain but less than the wild-type strain.

Then, the T6SS-dependent killing activity of MFE01 against *P. atrosepticum* was assessed (Fig. 2B). In case of competition with *P. atrosepticum* during 4 h at 28°C, the WT+EV strain reduced the quantity of surviving *P. atrosepticum* by three logs, while the Δ*gacS*+EV mutant did not cause a decrease in *P. atrosepticum* survival. In the Δ*gacS+gacS* mutant, a partial restoration of competition was observed with a 1-log reduction in the quantity of surviving *P. atrosepticum* (Fig. 2B). The presence of T6SS sheaths in MFE01 WT and in the Δ*gacS* mutant was visualized by fluorescence microscopy through the introduction of the plasmid pJN105:*tssB-sfgfp* containing a translational fusion between *tssB* and *sfgfp*, allowing fluorescent labeling of the T6SS sheath without preventing its contraction and recycling (18). In the Δ*gacS* mutant, no T6SS sheaths were observable, whereas they were clearly visible as bright lines crossing the cells in the wild-type strain (Fig. 2C).

To explore potential evidences of direct regulation of T6SS by Gac/Rsm system, we searched for the consensus pattern of RsmA binding sites on predicted mRNA, as described by Chihara et al. (28). Our search was focused on the 100 bp before the starting codon on the putative mRNA of *tssA*, a gene from the T6SS cluster. One compatible sequence was found located 72 bp upstream of putative ribosome binding site (RBS) (Fig. S10). The same method was used to search for potential RsmA binding sites in putative mRNAs from the *undA* and *hcnABC* operons (which synthesize 1-undececene and HCN, respectively). A sequence compatible with a potential RsmA binding was found 17 bp upstream of the putative RBS of the operon *undA* mRNA and another 26 bp upstream of the putative RBS of the operon *hcnABC* mRNA (Fig. S10). In addition, we searched for the consensus pattern of Rsm protein binding sites in predicted mRNA as characterized by Duss et al.(29). This motif was found to overlap (*undA* and *hcnABC* mRNAs) or be adjacent (*tssA* mRNA) to putative RBS.

### The deletion of *gacS* significantly impacts the emission of volatile compounds (VCs) in MFE01 and its competitivity against some pathogens

**Fig 3:**
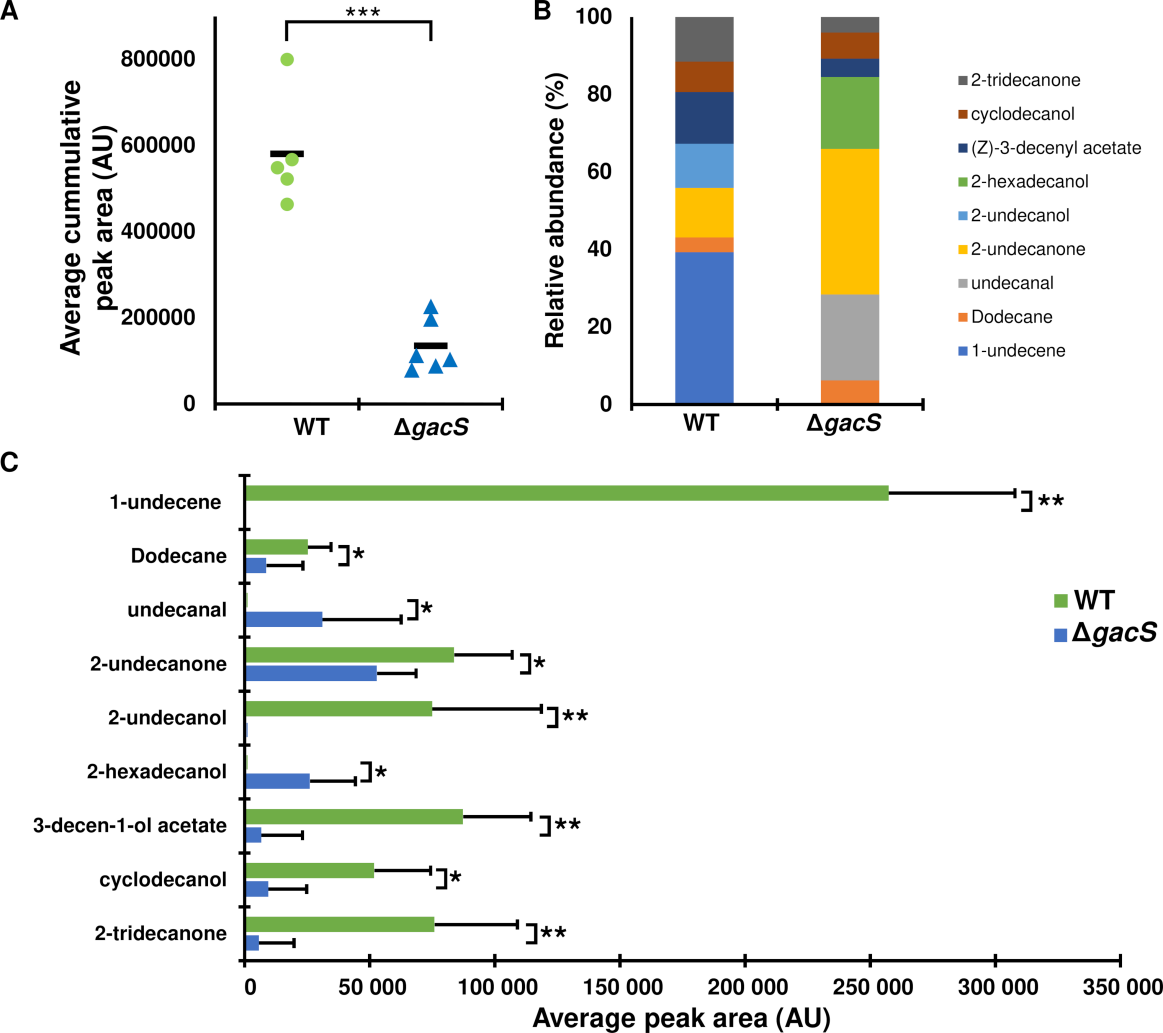
Deletion of the *gacS* gene disrupts the emission of volatile organic compounds in *P. fluorescens* MFE01. Volatile organic compounds emitted on LBG in 24 h by 40 µL of bacterial suspension (OD_580_ ≈ 1) of the wild-type strain (WT) or *gacS* gene deletion mutant (Δ*gacS*) analyzed by gas chromatography coupled with mass spectrometry (Headspace SPME GC/MS). The area under each chromatographic peak corresponding to a VOC was integrated for quantification. (**A**) Overall accumulation of VOCs in the headspace of MFE01 WT and Δ*gacS*. Each point corresponds to a different biological replicate. Green circles correspond to the wild-type strain, and blue triangles to the Δ*gacS* mutant. The black bars represent the mean. (**B**) Proportion of each VOC in volatilome of MFE01 WT and Δ*gacS*. (**C**) Comparison of the emitted quantity of each VOC by MFE01 (green bars) and Δ*gacS* (blue bars). These data correspond to the means of at least five independent trials (*n* ≥ 5). Error bars represent standard deviations. The quantities of emitted VOCs are compared using a non-parametric Mann–Whitney test. A *P*-value < 0.05 indicates statistically significant differences. *, *P* < 0.05; **, *P* < 0.01; ***, *P* < 0.001.

To measure the impact of GacS on volatile organic compound (VOC) emission, the volatilome of MFE01 was analyzed by trapping and analyzing VOCs emitted and accumulated in a vial over 24 h from either the WT strain or the Δ*gacS* mutant using headspace solid-phase microextraction (SPME) coupled with gas chromatography-mass spectrometry (GC/MS). Overall, the Δ*gacS* mutant emitted a total amount of VOCs that is 4.7 times lower than that of the WT strain (Fig. 3A). A more detailed analysis of their volatilomes revealed strong differences in both the diversity and abundance of the emitted compounds (Fig. 3B). While the WT strain predominantly emitted 1-undecene (approximately 40% of the volatilome), this compound was no longer emitted by the Δ*gacS* mutant (Fig. 3B and C). Similarly, 2-undecanol was no longer emitted upon deletion of the *gacS* gene (Fig. 3B and C). Thus, GacS exerts a strict positive control on 1-undecene and 2-undecanol emission. Furthermore, the Δ*gacS* mutant emitted undecanal and 2-hexadecanol, which were not detected among the VOCs emitted by the wild-type strain (Fig. 3B and C), suggesting that GacS is a negative regulator for the emission of these two VOCs. The amount of other VOCs, including 2-tridecanone and 2-undecanone, was only partially reduced in Δ*gacS*, implying that while GacS is not essential for their production, it exerts partial positive regulation on their emission.

**Fig 4:**
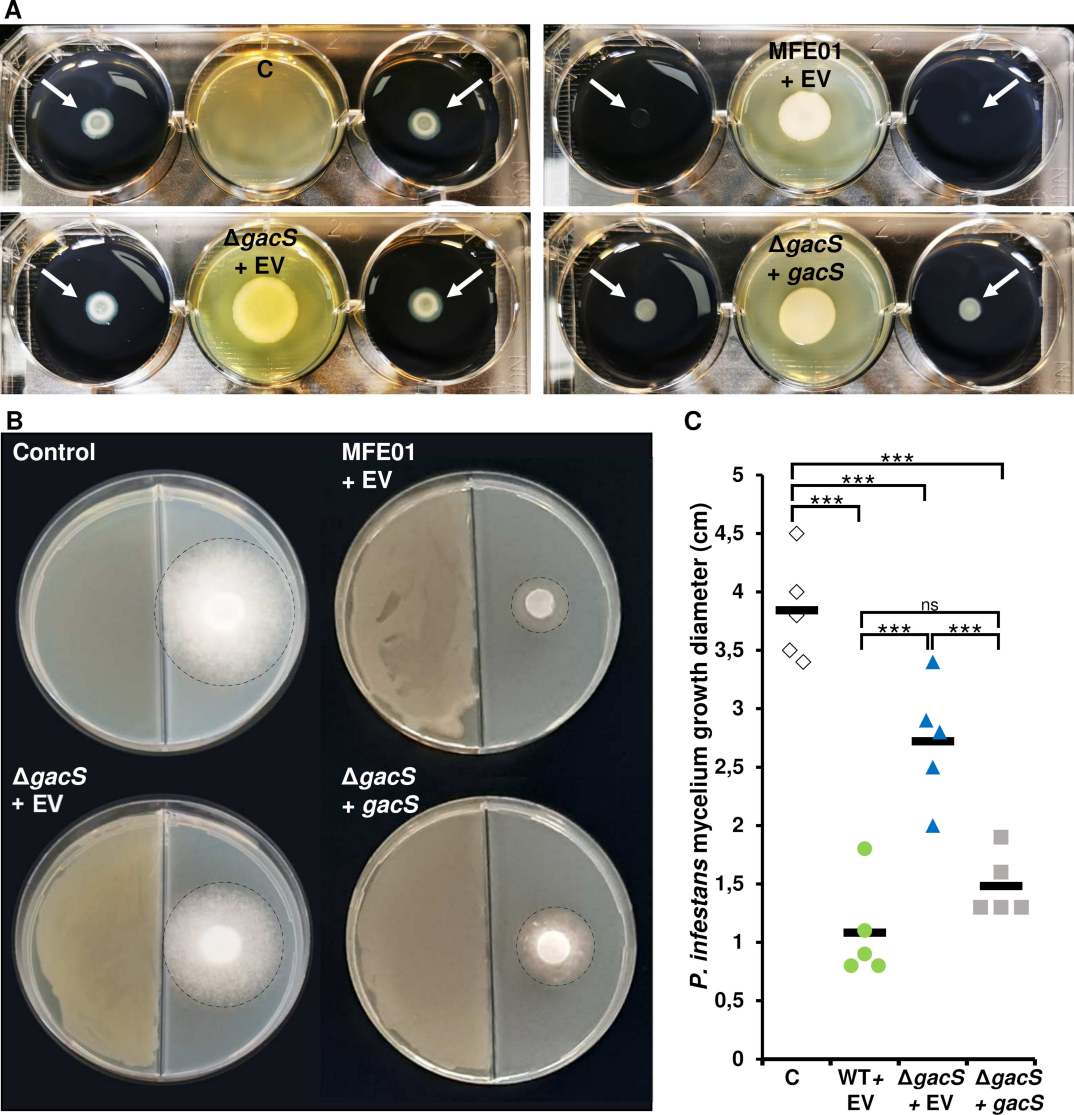
The *gacS* gene is involved in the aerial competition of MFE01. (**A**) The anti-*Legionella* activity of strains MFE01 and mutants. *Legionella pneumophila* Lens (outer wells of six-well plates) is cultured for 4 days in the presence of volatile molecules emitted by *P. fluorescens* MFE01or its mutants (central wells). After incubation at 28°C, the growth on agar of *L. pneumophila* Lens is visually evaluated. White arrows indicate the initial deposition of *L. pneumophila* Lens. (**B**) Representative photograph of the growth of *Phytophtora infestans* (right compartment) exposed to VCs from different MFE01 mutants (left compartment of bi-compartment Petri dishes) during 7 days of incubation at room temperature (21°C). The mycelium is delineated by a dashed circle. (**C**) Measurement of the mycelial growth diameter of *P. infestans* under the different conditions. Each point corresponds to a different biological replicate. White diamonds: *P. infestans* cultured in the absence of bacterial VCs. Green circles: *P. infestans* cultured in the presence of VCs emitted by MFE01 WT + EV. Blue triangles: *P. infestans* cultured in the presence of VCs emitted by MFE01 Δ*gacS*+EV. Gray squares: *P. infestans* cultured in the presence of VCs emitted by MFE01 Δ*gacS+gacS*. The black bars represent the means of each condition. These data correspond to five independent trials (*n* = 5). Quantitative data are compared using an ANOVA test. A *P*-value < 0.05 indicates statistically significant differences. ns, non-significant; ***P < 0.001. Wild-type MFE01 with the empty vector pJN105 (WT + EV), *gacS* mutant with the empty vector pJN105 (Δ*gacS*+EV) or with the vector pJN105 containing the *gacS* gene (Δ*gacS+gacS*). The negative control C corresponds to an experimental condition where *L.pneumophila* or *P. infestans* were grown in the absence of volatile molecules emitted by a bacterium.

The impact of *gacS* deletion was assessed on MFE01’s ability to inhibit, through its gas phase, the bacterium *Legionella pneumophila*, the causative agent of Legionnaires' disease. The inhibition of *L. pneumophila* was affected by the *gacS* deletion. Indeed, while the volatile molecules from the WT + EV strain completely inhibited the growth of *L. pneumophila*, its growth in the presence of volatiles compounds (VC) from the Δ*gacS*+EV strain was equivalent to that observed in the absence of VCs (control condition). The trans-expression of the wild-type *gacS* gene in the Δ*gacS* mutant (strain Δ*gacS+gacS*) lead to a decrease in *L. pneumophila* growth, thus partially restoring the phenotype (Fig. 4A).

The genome of MFE01 contains the *hcn*ABC genes involved in hydrogen cyanide (HCN) synthesis. The emission of HCN is described in some bacteria as being responsible for inhibiting the growth of the oomycete *Phytophtora infestans*, the causative agent of late blight. Some studies showed that the synthesis of HCN would be regulated by the GacA/GacS system (13). So, we assessed the ability of MFE01 and its *gacS* mutant to inhibit, through gas phase, *Phytophthora infestans*. After 7 days of growth on pea medium, the average diameter of *P. infestans* mycelial growth was 3.8 cm. When exposed to the VCs emitted by the WT + EV strain, the growth decreased to 1 cm. Exposure to the VCs from the Δ*gacS* + EV mutant and the Δ*gacS + gacS* mutant affected the mycelial growth, resulting in diameters of 2.7 cm and 1.48 cm, respectively (Fig. 4B and C). The *P. infestans* growth was fully inhibited by exposure to the VCs emitted by the non-transformed WT strain (Fig. S9). Furthermore, a mutant that no longer emitted 1-undecene, the Δ*undA* mutant, also completely inhibited the development of *P. infestans* (Fig. S9).

### HCN emission is impacted by *gacS* deletion and implicated in *Phytophthora infestans* growth inhibition

**Fig 5:**
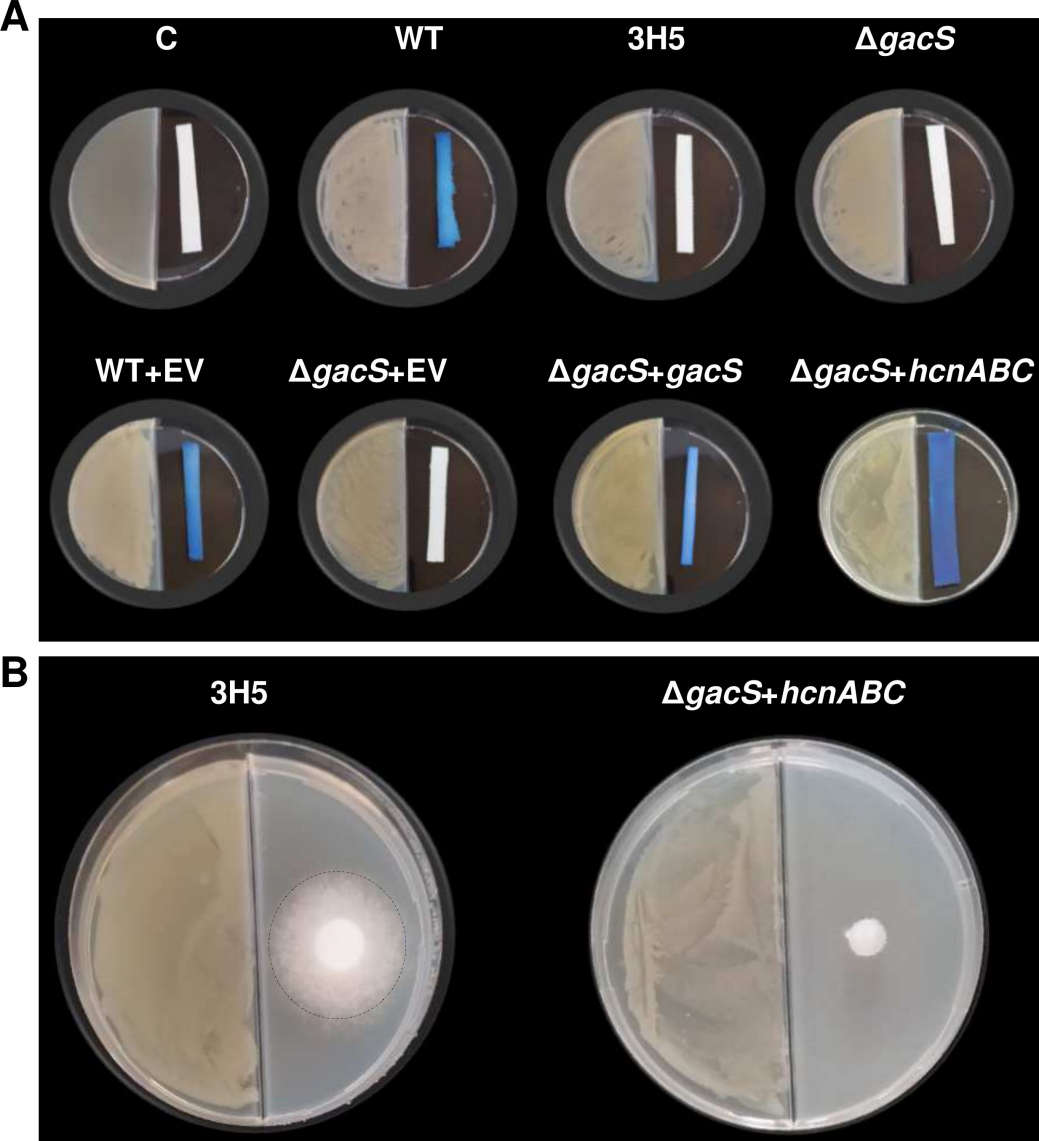
HCN is involved in the aerial competition of MFE01. (**A**) HCN emission by MFE01 or its mutants. When HCN is emitted by the bacteria (left side etof the two-compartment dishes) during 24 h at 28°C, a blue color forms on a strip impregnated with copper(II) ethylacetoacetate and 4,4-methylenebis(N,N-dimethylaniline) (right), highlighting the production of HCN qualitatively. C: negative control without bacteria, (WT): MFE01, (WT + EV): MFE01 with the empty vector pJN105, (Δ*gacS* +E V): *gacS* mutant with the empty vector pJN105, (Δ*gacS + gacS*): *gacS* deletion mutant with the vector pJN105 containing the *gacS* gene, (Δ*gacS + hcnABC): gacS* mutant with the vector pJN105 containing the *hcnABC* genes. (**B**) Representative photograph of the growth of *P. infestans* (right compartment) exposed to 3H5 or *gacS* mutant with the vector pJN105 containing the *hcnABC* genes (Δ*gacS + hcnABC)* (left compartment of bi-compartment Petri dishes) after 7 days of incubation in the presence of volatile molecules emitted by bacteria at room temperature (21°C). The mycelium is delineated by a dashed circle.

Based on a previous publication describing HCN as an inhibitor of *P. infestans* growth (30), a qualitative test was used to study the emission of HCN by MFE01 and its different mutants. MFE01 emitted enough HCN in 24 h at 28°C to allow the development of a blue color on a strip impregnated with copper(II) ethylacetoacetate and 4,4-methylenebis(N,N-dimethylaniline) (Fig. 5A). Neither the *gacS* mutant nor the 3H5 mutant allowed the coloration of the strip, indicating a reduction or absence of detectable HCN production in these two mutants.

Trans-expression in the *gacS* mutant of *hcnABC* genes that allows HCN synthesis from glycine restored detectable HCN production. The effect of the volatile phase of the 3H5 mutant, which produces little to no HCN, as well as that of the *gacS* mutant overexpressing the *hcnABC* genes was measured on the growth of *P.infestans* (Fig. 5B). After 7 days of growth on pea medium, the average diameter of *P. infestans* mycelial growth exposed to the VCs emitted by the 3H5 mutant (2.6 cm) is similar to that obtained with the *gacS* mutant. Upon exposure to the VCs emitted by *gacS* mutant overexpressing the *hcnABC* genes, the *P. infestans* growth was fully inhibited.

## DISCUSSION

The Gac/Rsm global regulatory pathway stands as a cornerstone for controlling major transitions in both clinically and environmentally relevant *Pseudomonas*, such as motile/sessile lifestyle, primary/secondary metabolism, or replicative/infective behavior (2, 31). *In silico* analyses suggest that MFE01 possess a fully functional Gac/Rsm system. Putative genes encoding the GacS/GacA TCS proteins as well as the two connectors LadS and RetS are present. Genes encoding the small non-coding RNAs RsmX, Y, and Z, as well as three putative genes encoding proteins from the carbon storage regulator family (CsrA family), are also detectable in the genome sequence of MFE01. These genes correspond to homologs of *rsmA*, *rsmE*, and *rsmI* from other *Pseudomonas* sp. (8, 32–36). Furthermore, the numerous phenotypic changes observed upon *gacS* deletion in our study provide strong evidence that this system is fully functional and active. A comparative transcriptomic analysis of genes by RT-qPCR between the MFE01 WT strain and the 3H5 or Δ*trpE* mutants showed a significant decrease in the expression of genes encoding elements of the GacS/GacA TCS, notably the *gacS* gene, only in the 3H5 mutant. Therefore, we constructed a Δ*gacS* deletion mutant to observe its phenotypes and determine if they are equivalent to those of 3H5 described in our previous study (17). It appears that deletion of the *gacS* gene resulted in a total loss of T6SS activity and a modification of the pool of emitted volatile molecules. Notably, the emission of 1-undecene was abolished, and the overall quantity of emitted VOCs was reduced. These phenotypes correspond to those of the 3H5 mutant (17), although the effects on VOCs emission were more pronounced in the case of *gacS* deletion.

The Gac/Rsm system seems to be essential for the biocontrol potential of MFE01. This idea is supported by the *in silico* detection of potential RsmA binding sites upstream or overlapping putative mRNA RBS associated with *tssA*, *undA,* and *hcnABC* expression. This is consistent with studies on the impact of this system on the biocontrol capabilities of other *Pseudomonas* species, including biocontrol agents or plant pathogens (for a recent review see Ref. [31]). Indeed, the Δ*gacS* mutant of MFE01 loses the ability to kill or inhibit the growth of both the potato pathogens *P. atrosepticum* and *P. infestans* as well as the human pathogen *L.pneumophila*. The T6SS-dependent killing of *P. atrosepticum* by MFE01 is notably mediated by an amidase-type toxin (18). It has already been shown that the three T6SSs of *P. aeruginosa* PA14 are under the control of RsmA (7). However, our study is based on a different approach than that of Allsop et al. In their study on PA14, a mutation in *rsmA* activates T6SSs that are poor or not active in the wild-type strain, whereas in our study on MFE01, *gacS* deletion abolishes the activity of a hyperactive T6SS. Nevertheless, these two studies seem to converge and demonstrate that RsmA-like regulators negatively control the production of a functional T6SS in different *Pseudomonas* species.

The inhibition of *P. infestans* by *P. fluorescens* strains has been previously reported, notably by the team of Prof. Weisskopf (13, 37, 38). However, this is the first time that it is shown that the MFE01 strain can inhibit *P. infestans* through its volatile molecules. This oomycete is the agent of late blight causing significant agro-economic impacts and is less sensitive to 1-undecene than *L. pneumophila* (17, 38). Our works confirm that it is a cocktail of volatile molecules that is effective against *P. infestans* rather than a single molecule (38). Among the active molecules, HCN has shown the strongest effect on *P. infestans* growth (30). Since GacS is known to be a major regulator of HCN synthesis, we investigated its production in our different strains (9, 10). In *Pseudomonas*, the secondary metabolite HCN is synthesized by oxidative decarboxylation of glycine. This anabolic pathway is catalyzed by the HCN synthase (syn. glycine dehydrogenase, E.C. 1.4.99.5) encoded by the contiguous structural genes *hcnABC* (9, 12). Here, we demonstrate that MFE01 emits HCN, which synthesis is strongly regulated by the Gac/Rsm pathway, showing a positive correlation between *P. infestans* inhibition and HCN emission. To confirm the role of HCN in *P. infestans* inhibition, we expressed *in trans* the HCN synthase that produces HCN in the *ΔgacS* mutant without activation of other volatile compound emission. This HCN production restores the inhibition of *P. infestans* growth, proving the role of HCN in this inhibition, accordingly to Anand et al. (13). The robustness of the observed inhibition is particularly striking in our study. When exposed to the volatile molecules emitted by MFE01, *P. infestans* is no longer—or almost no longer—able to develop its mycelium. Moreover, the deletion of *gacS* only partially alleviates the inhibition of *P. infestans* despite the very significant decrease in emitted volatile molecules. In our conditions, the absence of 1-undecene emission in the *undA* mutant did not abolish the inhibition of our isolate of *P. infestans* although this compound was described as being very active against many strains of oomycetes (38). Thus, MFE01 appears to emit one or more anti-*Phytophthora* molecules, which may be active at low doses, and could be a promising agent in the fight against *P. infestans* by the emission of volatile molecules capable of inhibiting its growth.

MFE01 is a strain with unconventional bacterial communication. It is interesting to note that in this bacterium, both the T6SS and the emission of 1-undecene, which seem to serve as communication mechanisms, are strictly controlled by the Gac/Rsm pathway (24, 25). These results are consistent with observations made in several *Pseudomonas* strains belonging to *P. aeruginosa*, *Pseudomonas chlororaphis*, and *Pseudomonas syringae* species, in which the Gac/Rsm pathway regulates cell-to-cell quorum-sensing communication based on the synthesis and perception of *N*-acyl-homoserine lactone (AHL) signals (2, 39–41). The global modification of the VOC pool in a Δ*gacS* mutant has also been observed in other *Pseudomonas* strain*s* (6, 42). For example, Cheng et al. demonstrated the involvement of the GacS sensor kinase in the regulation of alkene (including 1-undecene) production in *P. fluorescens* SBW25, which in turn affects plant growth in a species-dependent manner (6). This result is compatible with the fact that VOCs, as well as AHLs signaling molecules, are derived from cellular metabolism (2, 19), which itself is controlled by the Gac/Rsm regulatory network. Accordingly, control of communication systems by the master Gac/Rsm network could therefore be a generalizable fact to most *Pseudomonas*.

The ligand of GacS remains unknown, which leads to the exploration of a *gacS* transcription inhibition as a potential strategy for controlling the virulence of Gac/Rsm-dependent organisms (43). For example, an extract of *Bacillus* sp. BR3 has been observed to decrease the virulence of *P. syringae pv*. Tomato DC3000 by inhibiting *gacS* transcription, and consequently that of *rsmZ* and *rsmY* (43). However, the underlying mechanism behind this inhibition of *gacS* transcription in this study has not been elucidated. To date, the only reported mechanism for regulating *gacS* transcription is an unexplained positive feedback loop (43, 44).

Our study might suggest that the *gacS* transcription in MFE01 may be influenced by the synthesis of one or more VOCs. Specifically, in the 3H5 mutant, we observed reduced transcription of the *gacS* gene along with decreased levels of different VOCs, including 1-undecene (17). Notably, the Δ*undA* mutant, which no longer emits 1-undecene, still maintains an active T6SS and thus a functional Gac/Rsm system (25), indicating that 1-undecene is not directly involved in the regulation of *gacS* transcription. Hence, it is plausible that the other VOCs could serve as activators of transcriptional regulation of *gacS*. Modulating the emission of these VOCs by *gacS* and activating *gacS* transcription by one of them might help to elucidate the positive feedback loop of the *gacS* gene previously demonstrated (44). Thereby, this investigation may lead to identify novel targets for controlling the Gac/Rsm pathway. To the best of our knowledge, the hypothesis of GacS/GacA system regulation by a volatile molecule has not been investigated in details.

To conclude, this study provides compelling evidence that the Gac/Rsm system exerts significant control over the emission of VOCs and the activity of the T6SS in *P. fluorescens* MFE01. Thus, we propose that VOCs could act as potential positive activators of *gacS* transcription. This hypothesis holds promise for unveiling novel mechanisms governing the Gac/Rsm system, with potential implications for the development of anti-virulence strategies with significant agronomic and medical relevance.

## MATERIALS AND METHODS

### Strains, plasmids, culture conditions, and chemicals

The strains and plasmids used in this study are listed in Table S1. *P. fluorescens, P. aeruginosa, E. coli*, and *P. atrosepticum* strains were cultured in lysogeny broth (LB) medium, while *L. pneumophila* Lens strain was cultured in buffered yeast extract (BYE) medium or on charcoal-buffered yeast extract agar (BCYE) plates (45). *P. fluorescens* strains were grown at 28°C, *P. atrosepticum* at 25°C, *P. aeruginosa*, *E. coli*, and *L. pneumophila* Lens strains at 37°C. All liquid cultures were maintained under constant rotary agitation at 180 rpm. Antibiotics were added to the medium as needed: 15 µg/mL tetracycline for *P. atrosepticum* and 20 or 50 µg/mL gentamicin for *E. coli* or *P. fluorescens*, respectively. Cultures of strains containing the pJN105 plasmid were supplemented with 0.02% (w/v) L-arabinose to mildly induce gene expression (46). All chemicals were obtained from Sigma-Aldrich (St. Louis, USA), unless otherwise stated.

*P. infestans* oomycete was cultured on pea agar prepared as described by Puopolo et al. with slight modifications (25% frozen peas and 15% agar) (47). A 6 mm diameter agar disk with growing mycelium harvested from a pre-existing culture of *P. infestans* was deposited on the pea agar to initiate a new culture as described by De Vrieze et al. (48).

### *In silico* analyses of GacA/GacS pathway in MFE01

Each gene was identified by sequence alignment using BLAST software from National Center for Biotechnology Information site and via the pseudomonas.com database, using *P. aeruginosa* PAO1, *P. fluorescens* SBW25, *P. ogarae* F113, *P. protegens* Pf-5, and *P. protegens* CHA0 as reference strains. Tblastn was used to identify the coding sequences of GacS, GacA, LadS, RetS, and CsrA-like protein. Blastn was used to identify the Rsm-like non-coding RNAs (ncRNAs) genes. If an analysis yielded multiple results, only the one with the lowest e-value was selected. For CsrA-like proteins and Rsm ncRNA, the name of the protein or ncRNA of the reference strain offering the best e-value was retained. Alignments were performed using the NCBI BLAST software to obtain percent identity score and query cover between sequences. The ncRNAs of *P. fluorescens* SBW25 and *P. protegens* CHA0 are not specified as they are not annotated in the reference genome. For ncRNAs, results with query cover lower than 25% were considered as no significant.

### General molecular biology procedures

PCR reactions were performed following standard procedures using Phusion high-fidelity DNA polymerase (New England Biolabs [NEB], Ipswich, USA) in high-fidelity buffer. The annealing temperature of primers was determined using the NEB Tm calculator (https://tmcalculator.neb.com/#!/main). Primers specified in Table S2 were obtained from Eurogentec, Belgium. Use of restriction enzymes (NEB) and T4 DNA ligase (NEB) followed manufacturer’s guidelines. Genomic DNA extraction and purification were performed using the GeneJET Genomic DNA Purification Kit (Thermo Fisher, Waltham, USA). After PCR, product purification was carried out either with the PCR Purification Kit (Qiagen, Hilden, Germany) or the Gel Extraction Kit (Qiagen) for products migrated on agarose gel. Each kit was used according to the manufacturers instructions.

### Construction of MFE01 Δ*gacS* mutant strain

The scarless in-frame deletion mutant of the *gacS* gene, named Δ*gacS*, harbors a 2,523 bp deletion within the *gacS* gene. To generate this deletion, the upstream and downstream regions of *gacS* were individually amplified by PCR using the primer pairs M1-gacS/M2QC-gacS (888 bp) and M3QC-gacS/M4-gacS (776 bp), respectively. The resulting amplicons were used in an overlapping PCR and re-amplified using primers M1-gacS/M4-gacS. The region containing the deleted *gacS* gene thus generated was ligated end-to-end using T4 DNA ligase into the suicide vector pAKE604 digested with SmaI (49). The vector was then transformed into *E. coli* strain top 10 (Thermo Fisher Scientific). After confirmation by Sanger sequencing (Genewiz, Leipzig, Germany), the constructed plasmid was introduced into *E. coli* strain S17.1 (50). Subsequently, this plasmid was transferred into MFE01 by conjugation, and a double recombination event was selected following a previously described method (51). The deletion mutant of *gacS*, Δ*gacS*, was verified by PCR analysis and DNA sequencing.

### Insertion of *gacS* into pJN105 expression vector

The *gacS* gene was amplified using the gacS-F/gacS-XbaI-R primers. The amplified fragment (2832 bp) was digested with XbaI. The pJN105 vector was digested with XbaI and SmaI to generate a cohesive end and a blunt end. Subsequently, the *gacS* gene was cloned into the pJN105 plasmid (46), under the control of the L-arabinose-inducible promoter. The resulting pJN105:*gacS* plasmid was verified by sequencing.

### Insertion of *hcnABC* into pJN105 expression vector

The *hcnABC* genes were amplified using the *hcnABC*-EcoRI-F/*hcnABC*-XbaI-R primers. The amplified fragment (3,031 bp) was digested with XbaI and EcoRI. The pJN105 vector was digested with XbaI and EcoRI to generate cohesive ends. Subsequently, the *hcnABC* operon was cloned into the pJN105 plasmid, under the control of the L-arabinose-inducible promoter. The resulting pJN105:*hcnABC* plasmid was verified by sequencing.

### MFE01 transformation by electroporation

Fresh colonies of MFE01 or its mutants were resuspended in a cold sterile solution of 300 mM saccharose (Fisher Chemicals), underwent two washes, and then resuspended in 100 µL of 300 mM saccharose. These competent cells and 150 ng of plasmid were introduced into 1 mm spaced electroporation cuvettes (Fisher Scientific, Hampton, USA) and subjected to electroporation at 1.8 kV for 5 ms, 200 Ω resistance, and 25 µF capacitance. After electroporation, 700 µL of LB were added, and cells were regenerated for 1 h and 30 min at 28°C with agitation. Transformed bacteria were plated on LB agar supplemented with gentamicin.

### RNA extraction and RT-qPCR

Colonies of MFE01 WT or its 3H5 mutant were picked from six-well plates after 24 h of incubation in the presence of volatile molecules emitted by GFP-tagged *L. pneumophila* Lens. Bacteria were resuspended in 400 µL of buffer (12.5 mM Tris, 5 mM EDTA, and 10% glucose), 400 µL of saturated phenol (pH 4.6), and 0.4 g of glass beads (diameter, 0.2–0.3 mm; Sigma) before cell lysis using a Fastprep instrument (5.5 m/s, 30 s, twice; ThermoFisher Scientific, Illkirch, France). After centrifugation at 14,000×*g* for 5 min, 1 mL of Trizol reagent (ThermoFisher Scientific) was added to the supernatants. Samples were incubated at room temperature for 5 min. Total RNA was extracted twice with chloroform. RNA was precipitated with isopropanol, and the precipitate was washed with 75% ethanol. RNA was then dissolved in nuclease-free water, and contaminating DNA was removed using DNase I without RNase (Turbo DNA-free kit, ThermoFisher Scientific), following the manufacturer’s instructions. RNA concentration and purity were measured using the NanoDropTM 2000 spectrophotometer (NanoDrop Technologies, USA). RNA quality was verified using the Shimadzu MultiNA capillary electrophoresis system (Shimadzu, Marne-la-Vallée, France), following the manufacturer’s instructions. Samples were stored at −80°C until analysis. Total RNA was reverse-transcribed into complementary DNA (cDNA) using the GoScript Reverse Transcriptase kit (Promega) according to the manufacturer’s recommendations. Complementary DNA products were used for quantitative PCR (qPCR) to analyze the expression of the genes *gacS*, *gacA*, *rsmA*, *rsmE*, *ladS*, *retS*, and *recA*. qPCR was performed using the LightCycler FastStart DNA Master plus SYBR Green I kit in a LightCycler 480 (Roche Molecular Systems, Pleasanton, CA, USA). Reactions were prepared in a total volume of 10 µL containing 5 µL of SYBR 2× mix, 2 µL of water, 2 µL of diluted cDNA, and 0.5 µL of 10 µM primers. The PCR program consisted of an initial step at 95°C for 10 min, followed by 45 cycles at 95°C for 10 s, 60°C for 10 s, and 72°C for 10 s. An additional step from 65°C to 95°C (0.5 °C/s) was added to confirm that a single product was amplified in each reaction. A standard curve was obtained with serial dilutions of DNA extracted from the MFE01 strain to calculate amplification efficiencies for each gene. The Pfaffl method was used to assess the relative quantitative variation between strains (52). Expression levels *of gacS, gacA, rsmA, rsmE, ladS*, and *retS* were quantified relative to the reference gene *recA*. Gene expression of the wild-type strain MFE01 incubated at 28°C was used as a reference. RT-qPCR was performed in independent biological triplicates and two technical replicates.

### Supernatant protein extraction

Five milliliters of overnight culture adjusted to OD_580_ = 1 was centrifuged at 7,500×*g* for 5 min at room temperature. Then, 2 mL of the supernatant was filtered through a 0.22 µm Millipore membrαne (Merck). Trichloroacetic acid was added to reach a final concentration of 10% (w/v), and the mixture was incubated at 4°C overnight. The solution was then centrifuged at 13,000×*g* for 30 min at 4°C, and the supernatant was removed. The resulting precipitate was washed twice with 2 mL of cold 100% acetone (VWR Chemicals, Radnor, USA) and centrifuged at 13,000×*g* for 30 min at 4°C. Finally, the precipitate was air-dried for 30 min and resuspended with 20 µL of Laemmli buffer 2× (Nupage, Invitrogen, Waltham, USA) containing 5% β-mercaptoethanol. Each extraction was performed independently three times.

### SDS-PAGE analysis

Protein solutions were incubated for 5 min at 95°C, and proteins equivalent to 2 mL of the supernatant of a bacterial suspension equilibrated at OD_580_ = 1 were separated on a 12% bis-acrylamide gel (Biorad, Hercules, USA). Protein electrophoresis and visualization were then performed as previously described (51). Identification of Hcp by MALDI-TOF was performed according to the methods described in a previous study (53).

### Killing assays

Killing assays of *P. atrosepticum* strain by MFE01 were conducted according to previously described methods (51, 53, 54). The *P. atrosepticum* strain prey bacteria used in this study is the strain CFBP6276 +pME6000 :*luxR*-P*luxI::gfp-cfp* (55). Each experiment was performed four times.

### Visualization of T6SS sheaths

Strains were transformed with the plasmid pJN105 containing the translational fusion *tssB-sfGFP* before observation by fluorescence microscopy, following (18).

### HS-SPME/GC-MS

Emission of volatile organic compounds (VOCs) was analyzed according to procedures described in a previous study (25).

### Anti-*Legionella* activity tests

Inhibition of *L. pneumophila* by MFE01 or its mutants was tested using a qualitative airborne inhibition assay in a six-well plate, as previously described (45). Four independent trials were conducted for each experiment.

### Anti-*Phytophthora infestans* activity tests

Inhibition of *P. infestans* by MFE01 or its mutants was tested using an airborne inhibition assay in a two-compartment Petri dish. In the left compartment, 50 µL of overnight culture of MFE01 or mutants was spread on LB and incubated for 24 h at 28°C to obtain a fully colonized half of the Petri dish. After this period, a mycelium disk with a diameter of 6 mm taken from a pre-existing culture of *P. infestans* was placed on the pea agar in the second half of the Petri dish. Petri dishes were incubated at room temperature (~21°C) for 7 days before measuring the growth diameter. Each experiment was repeated five times.

### HCN detection

Wattmann paper strips were soaked in 10 mL of chloroform containing 50 mg copper(II) ethylacetoacetate and 50 mg 4,4-methylenebis(N,N-dimethylaniline) and were dried overnight at room temperature. These strips were placed in an empty compartment of two-compartment petri dishes, and the other compartment was filled with LB agar inoculated with 50 µL of bacterial overnight culture. Images were taken after 24 h of incubation at 28°C. Each experiment was repeated five times.

### Statistical analyses

The one-way ANOVA test was used to compare more than two groups (i.e., for *P. atrosepticum* recovery cell count and *P. infestans* growth diameter). The Tukey’s *post hoc* test was used to assess differences between each group. The Wilcoxon test was used to compare the relative expression of genes coding Gac/Rsm pathway proteins in 3H5 strain compared with the WT strain. The difference in VOC emission was compared using a Mann–Whitney test. In each experiment, *α*-risk was set to 0.05. Each experiment was performed at least three times.

## SUPPLEMENTAL MATERIAL

Tables S1 and S2 jb.00388-24-s0001.docx Strains, plasmids, and oligos used in this study.

Figures S1 to S10 jb.00388-24-s0002.pdf Gac/Rsm pathway, Gac/Rsm aligments, *P. infestans* inhibition, and RsmA binding sites.

ASM does not own the copyrights to Supplemental Material that may be linked to, or accessed through, an article. The authors have granted ASM a non-exclusive, world-wide license to publish the Supplemental Material files. Please contact the corresponding author directly for reuse.
