# Supplementary material for: The GacS/GacA two-component system strongly regulates antimicrobial competition mechanisms of Pseudomonas fluorescens MFE01 strain
Source: J Bacteriol. 2025 Jan 23;207(2):e00388-24. doi: 10.1128/jb.00388-24 (PMC11841057; doi:10.1128/jb.00388-24)
Supplement: Tables S1 and S2 — Strains, plasmids, and oligos used in this study. [file jb.00388-24-s0001.docx]

Supplementary table 1

| **Strains** | **Specific Characteristics** | **Reference/Source** |
| --- | --- | --- |
| ***Pseudomonas fluorescens*** |  |  |
| MFE01 (WT) | Environmental isolate, RifR. | Decoin et al., 2014 |
| MFE01 Δ*gacS* (Δ*gacS*) | MFE01 with central deletion in the *gacS* gene. | This study |
| MFE01+pJN105 (WT+EV) | MFE01 with empty vector pJN105, GmR. | Bouteiller et al., 2020 |
| MFE01Δ*gacS* + pJN105 (Δ*gacS+EV)* | MFE01 ΔgacS with empty vector pJN105, GmR. | This study |
| MFE01 Δ*gacS*+*gacS* (Δ*gacS*+*gacS*) | MFE01 ΔgacS with plasmid pJN105 carrying the wild-type *gacS* gene, GmR. | This study |
| MFE01 Δ*undA* (Δ*undA*) | MFE01 with a central deletion in the *undA* gene. | Dupont et al., 2023 |
| MFE01 3H5 (3H5) | Mini-Tn5 mutant of MFE01, *trpE::Tn5*. | Corre et al., 2021 |
| MFE01 Δ*trpE* (Δ*trpE*) | MFE01 with in frame deletion in the *trpE* gene. | Corre et al., 2021 |
| MFE01+pJN105 *tssB-gfp* | MFE01 with plasmid pJN105 carrying translational fusion *tssB-gfp* | This study |
| MFE01 Δ*gacS+* pJN105 *tssB-gfp* | Δ*gacS* mutant with plasmid pJN105 carrying translational fusion *tssB-gfp* | This study |
| MFE01 Δ*gacS+* pJN105 *hcnABC* | Δ*gacS* mutant with plasmid pJN105 carrying *hcnABC* genes | This study |
| ***Legionella pneumophila*** |  |  |
| CIP 108286 | Virulent serogroup 1 strain, Lens, gfp, CmR. | Bigot et al., 2013 |
| ***Pectobacterium atrosepticum*** |  |  |
| CFBP6276 + pME6000 :*luxR*-P*luxI*::*gfp-cfp* | Potato soft rot pathogen CFBP6276 transformed with plasmid pME6000 :*luxR-PluxI* ::*gfp-cfp*. TetR | Chane et al., 2019 |
| ***Escherichia coli*** |  |  |
| Top10® | *F- mcrA ∆(mrr-hsdRMS-mcrBC) Φ80lacZ∆M15 ∆lacX74 recA1 araD139 ∆(araleu)7697 galU galK rpsL (StrR) endA1 nupG.* | ThermoFischer Scientific |
| S17.1 | RP4-2-*Tc::Mu, aph::Tn7, recA*, SmR, donor strain for conjugation. | Simon et al, 1983 |
| ***Phytophtora infestans*** |  |  |
| 20096 | Potato late blight pathogen. | FN3PT collection |
| **Plasmids** | **Specific Characteristics** | **Reference/Source** |
| pAKE604 | Conjugative suicide vector, *oriT*, *lacZ,* *sacB*, ApR, KmR. | EI-Sayed et al., 2001 |
| pAKE604+Δ*gacS* | Plasmid pAKE604 carrying the sequence used for the in-frame deletion of the *gacS* gene. | This study |
| pJN105 | Arabinose-induced cloning vector, derived from pBBR1-MCS5, GmR. | Newman et al., 1999 |
| pJN105+*gacS* | Plasmid pJN105 carrying the *gacS* gene under the control of an arabinose-inducible promoter. GmR. | This study |
| pJN105 *hcnABC* | Plasmid pJN105 carrying the *hcnABC* genes under the control of an arabinose-inducible promoter. GmR. | This study |
| pME6000 | Cloning vector, derived from pBBR1-MCS, TcR. | Maurhofer et al., 1998 |
| pME6000:*luxR-PluxI::gfp-cfp* | Plasmid pME6000-*cfp* with a transcriptional fusion *PluxI::gfp-cfp* under the control of *luxR* expression. TcR. | Chane et al., 2019 |

Supplementary table 2

| **Mutagenesis primers** | **Sequences (5’ to 3’)** |
| --- | --- |
| M1-*gacS* | GTAACCGATGGCTTTGAGTT |
| M2QC-*gacS* | ATACTTCGTGGGTCCAGGTGATGAGACCTTTGATTCCCAGTTTC |
| M3QC-*gacS* | TCATCACCTGGACCCACGAAGTATACAGCCACGACCAGAATG |
| M4-*gacS* | CGCGATTGATCAGGTGCTTG |
| **Overexpression primers** | **Sequences (5’ to 3’)** |
| *gacS*-F | AGGAGG*AAAAACAT*ATGTGTCAACAGATCAGCG |
| *gacS*-XbaI-R | TGC*TCTAGA*CGACAACTGTAGCGCTTT |
| *hcnABC*-EcoRI-F | CCG*GAATTC*AGGAGGACACCGTAATGAACTGCCTAG |
| *hcnABC*-XbaI-R | TGC*TCTAGA*TCACTGACAGATTGCATTCG |
| **qPCR primers** | **Sequences (5’ to 3’)** |
| qRT-PCR-*gac*S-F | AGTCCGGTCCACTTCAACAC |
| qRT-PCR- *gac*S-R | GCATGGACGACTACCTGACC |
| qRT-PCR-*gac*A-F | TTCGGTGACAGGCACAACTT |
| qRT-PCR- *gac*A-R | AACCAATGATTCGCCGTTCG |
| qRT-PCR-*rsm*A-F | ACCTCTTTCGGAGCGTTGAC |
| qRT-PCR- *rsm*A-R | CTGATTATCGGTGACGGCGA |
| qRT-PCR-*rsm*E-F | ATCACGATCACCATCCTCGG |
| qRT-PCR- *rsm*E-R | CCACGTTTTTCGGAGCGTTG |
| qRT-PCR-*lad*S-F | AGCAAGTCGCTGGACTTCAA |
| qRT-PCR- *lad*S-R | ATTGTCCAGCAGGCATTCCA |
| qRT-PCR-*ret*S-F | CTGGCAGCGAAGATCAAGGA |
| qRT-PCR- *ret*S-R | TTGATTGAGTTCGTCCGCCA |
