# Supplementary material for: The GacS/GacA two-component system strongly regulates antimicrobial competition mechanisms of Pseudomonas fluorescens MFE01 strain
Source: J Bacteriol. 2025 Jan 23;207(2):e00388-24. doi: 10.1128/jb.00388-24 (PMC11841057; doi:10.1128/jb.00388-24)
Supplement: Figures S1 to S10 — Gac/Rsm pathway, Gac/Rsm aligments, P. infestans inhibition, and RsmA binding sites. [file jb.00388-24-s0002.pdf]

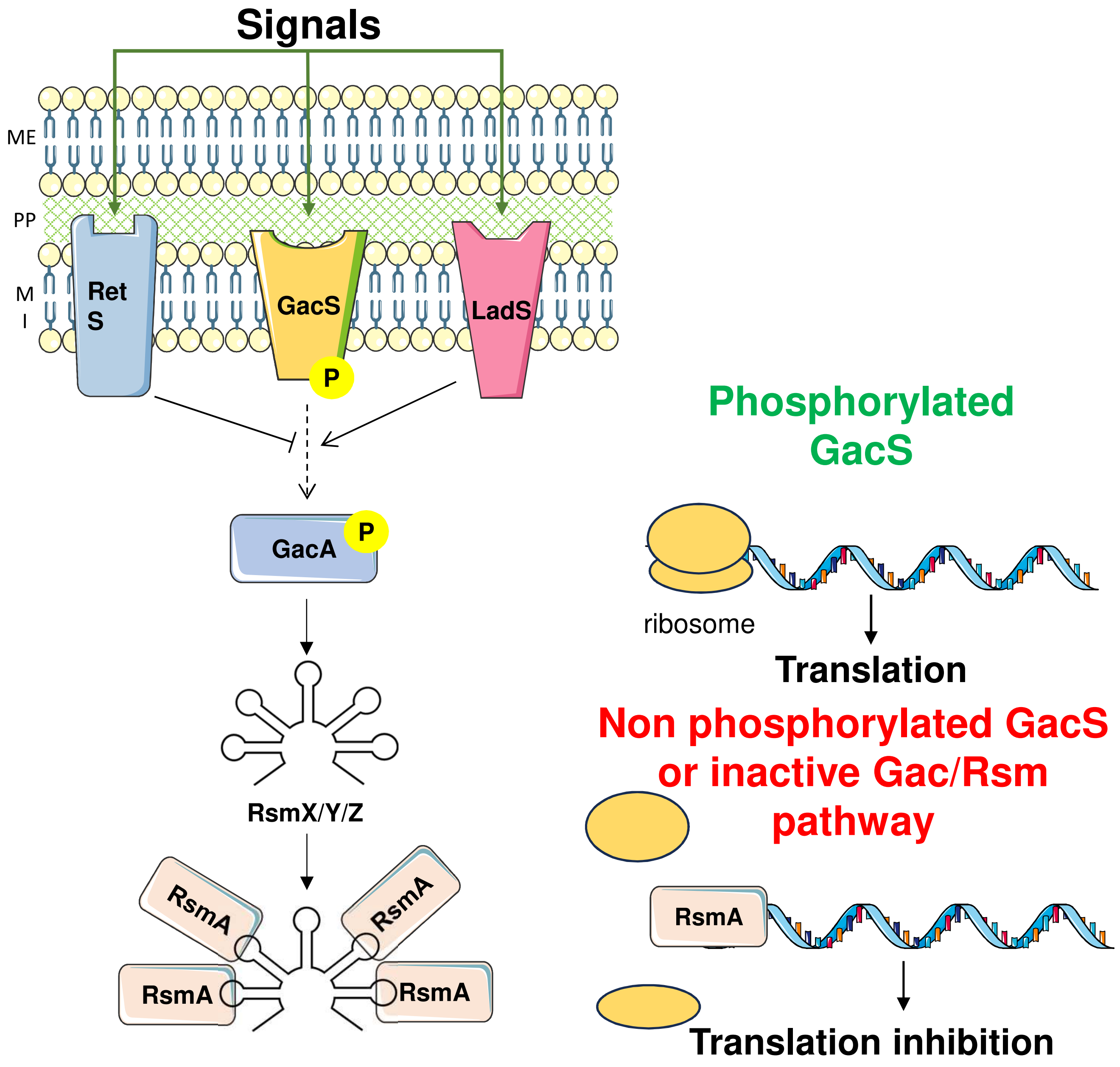

Supplementary Figure 1: Molecular operation of the Gac/Rsm system in *Pseudomonas.*

The GacS protein, the sensing component of the system, is a histidine kinase anchored in the inner membrane by two transmembrane domains. The interaction between GacS at its N-terminal domain and its ligand, still unknown to date, induces its ATP-dependent autophosphorylation. The generated phosphate is transmitted via a phospho-relay to the second component of the system, the cytoplasmic response regulator GacA. In parallel, the two connectors RetS and LadS can respectively negatively or positively modulate the phosphorylation of GacA by GacS. GacA activates the expression of small non-coding RNAs RsmX, Y, Z, capable of sequestering proteins from the RsmA family, allowing the translation of RsmA target mRNAs. Conversely, if RsmA is free, it binds to the 5' end of specific mRNAs and prevents their translation.

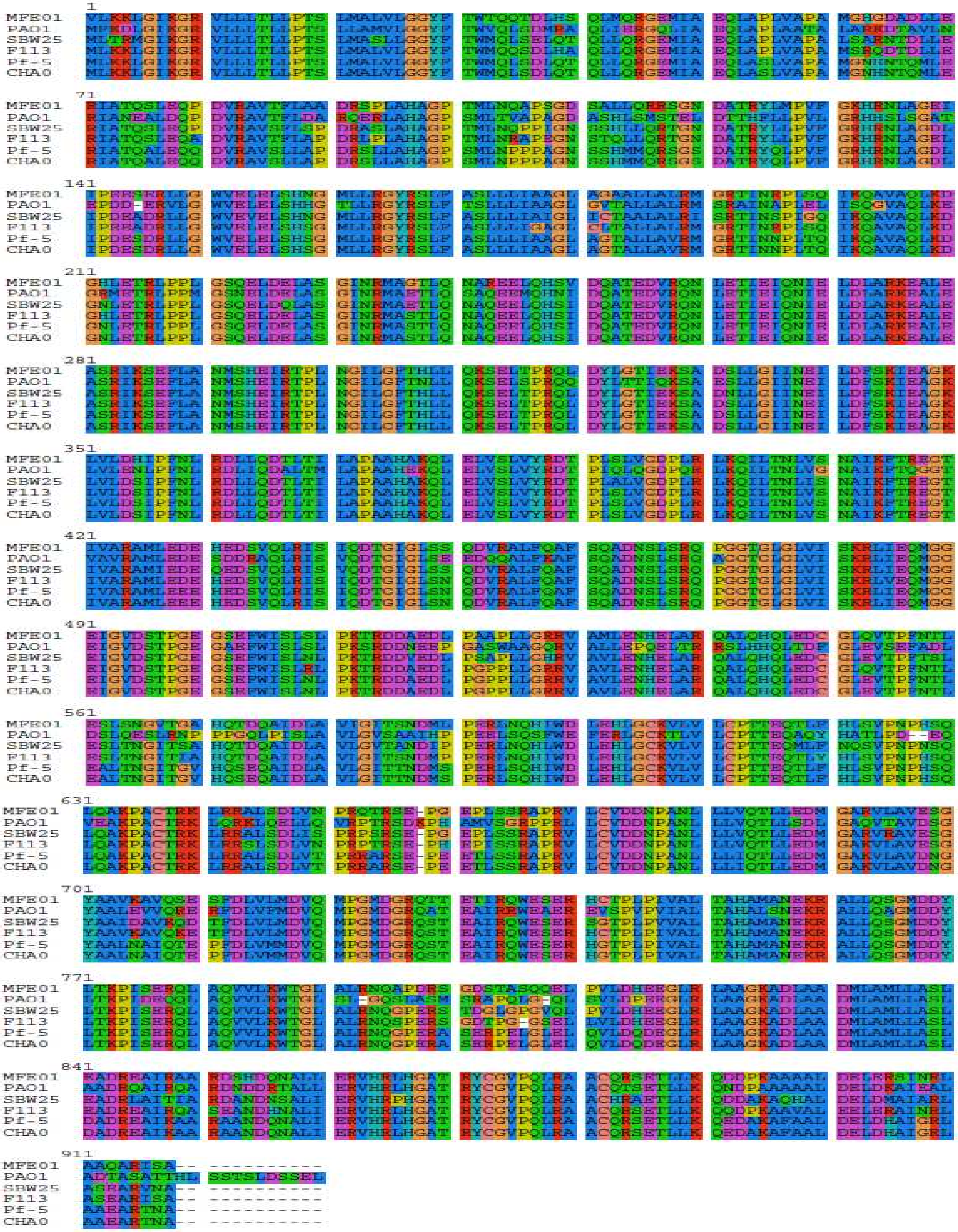

Supplementary Figure 2: Alignment of GacS protein sequences from *Pseudomonas fluorescens* MFE01 with those from different *Pseudomonas*. Except for MFE01, the sequences were retrieved from the "*Pseudomonas* genome database." MFE01: *Pseudomonas fluorescens* MFE01. PAO1: *Pseudomonas aeruginosa* PAO1. SBW25: *Pseudomonas fluorescens* SBW25. F113: *Pseudomonas ogarae* F113. Pf-5: *Pseudomonas protegens* Pf-5. CHA0: *Pseudomonas protegens* CHA0

```
        1
MFE01   LIRVLVVDDH DLVRTGITRM LADIDGLQVV GQAESGEESL IKARELKPDV VLMDVKMPGI GGLEATRKLL
PA01    MIKVLVVDDH DLVRTGITRM LADIEGLQVV GQADCGEDCL KLARELKPDV VLMDVKMPGI GGLEATRKLL
SBW25   MIRVLVVDDH DLVRTGITRM LADIDGLQVV GQAESGEESL IKARELKPDV VLMDVKMPGI GGLGATTKLL
F113    MIRVLVVDDH DLVRTGITRM LADIDGLQVV GQAESGEESL IKARELKPDV VLMDVKMPGI GGLEATRKLL
Pf-5    MIRVLVVDDH DLVRTGITRM LADIDGLQVV GQAESGEESL LKARELKPDV VLMDVKMPGI GGLEATRKLL
CHA0    MIRVLVVDDH DLVRTGITRM LADIDGLQVV GQAESGEESL LKARELKPDV VLMDVKMPGI GGLEATRKLL

       71
MFE01   RSHPDIKVVA VTVCEEDPFP TRLLQAGAAG YLTKGAGLPE MVQAIRLVFA GQRYISPQIA QQLAIKSFQP
PA01    RSQPDIKVVV VTVCEEDPFP TRLMQAGAAG YMTKGAGLEE MVQAIRQVFA GQRYISPQIA QQLALKSFQP
SBW25   RSHPDIKVVV VTVCEEDPFP TRLLQAGAAG YLTKGAGLAE MVQAIRLVFA GQRYISPQIA QQLAIKSFQP
F113    RSHPDIKVVA VTVCEEDPFP TRLLQAGAAG YLTKGAGLNE MVQAIRLVFA GQRYISPQIA QQLAIKSFQP
Pf-5    RSHPDIKVVA VTVCEEDPFP TRLLQAGAAG YLTKGAGLNE MVQAIRLVFA GQRYISPQIA QQLVFKSFQP
CHA0    RSHPDIKVVA VTVCEEDPFP TRLLQAGAAG YLTKGAGLNE MVQAIRLVFA GQRYISPQIA QQLVFKSFQP

      141
MFE01   -TNDSPFDAL SEREIQIALM IVGCQKVQII SDKLCLSPKT VNTYRYRIFE KLSISSDVEL TLLAVRHGMV
PA01    QQHDSPFDSL SEREIQIALM IANCHKVQSI SDKLCLSPKT VNTYRYRIFE KLSITSDVEL ALLAVRHGMV
SBW25   -TSDSPFDAL SEREIQIALM IVGCQKVQTI SDKLCLSPKT VNTYRYRIFE KLAISSDVEL TLLAVRHGMV
F113    -TNDSPFDAL SEREIQIALM IVGCQKVQII SDKLCLSPKT VNTYRYRIFE KLSISSDVEL TLLAVRHGMV
Pf-5    -SSDSPFDAL SEREIQIALM IVGCQKVQII SDKLCLSPKT VNTYRYRIFE KLSISSDVEL TLLAVRHGMV
CHA0    -SSDSPFDAL SEREIQIALM IVGCQKVQII SDKLCLSPKT VNTYRYRIFE KLSISSDVEL TLLAVRHGMV

      211
MFE01   DASA
PA01    DAAS
SBW25   DASA
F113    DASA
Pf-5    DASL
CHA0    DASL
```

Supplementary Figure 3: Alignment of GacA protein sequences from *Pseudomonas fluorescens* MFE01 with those from different *Pseudomonas*. Except for MFE01, the sequences were retrieved from the "*Pseudomonas* genome database." MFE01: *Pseudomonas fluorescens* MFE01. PAO1: *Pseudomonas aeruginosa* PAO1. SBW25: *Pseudomonas fluorescens* SBW25. F113: *Pseudomonas ogarae* F113. Pf-5: *Pseudomonas protegens* Pf-5. CHA0: *Pseudomonas protegens* CHA0

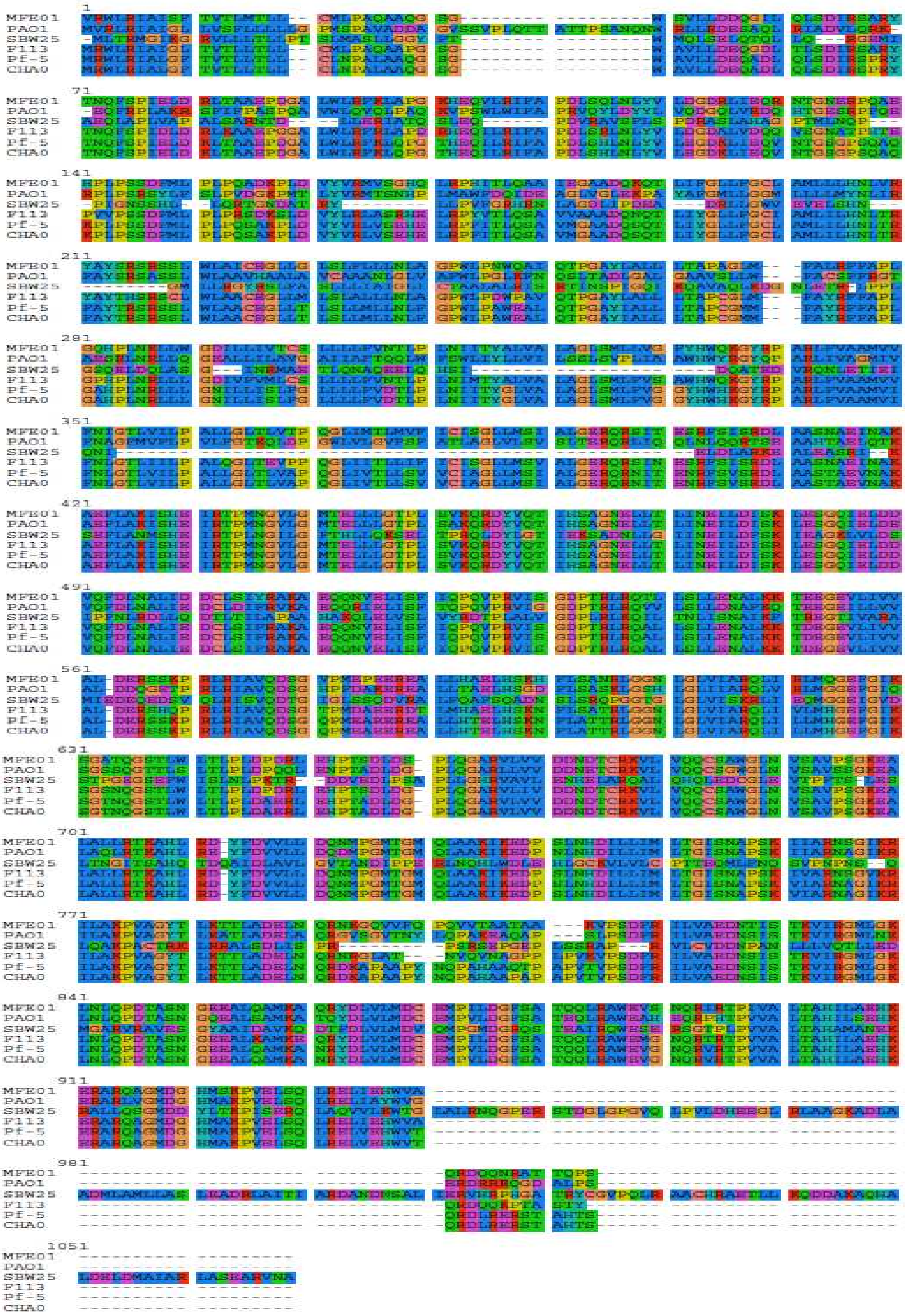

Supplementary Figure 4: Alignment of RetS protein sequences from *Pseudomonas fluorescens* MFE01 with those from different *Pseudomonas*. Except for MFE01, the sequences were retrieved from the "*Pseudomonas* genome database." MFE01: *Pseudomonas fluorescens* MFE01. PAO1: *Pseudomonas aeruginosa* PAO1. SBW25: *Pseudomonas fluorescens* SBW25. F113: *Pseudomonas ogarae* F113. Pf-5: *Pseudomonas protegens* Pf-5. CHA0: *Pseudomonas protegens* CHA0

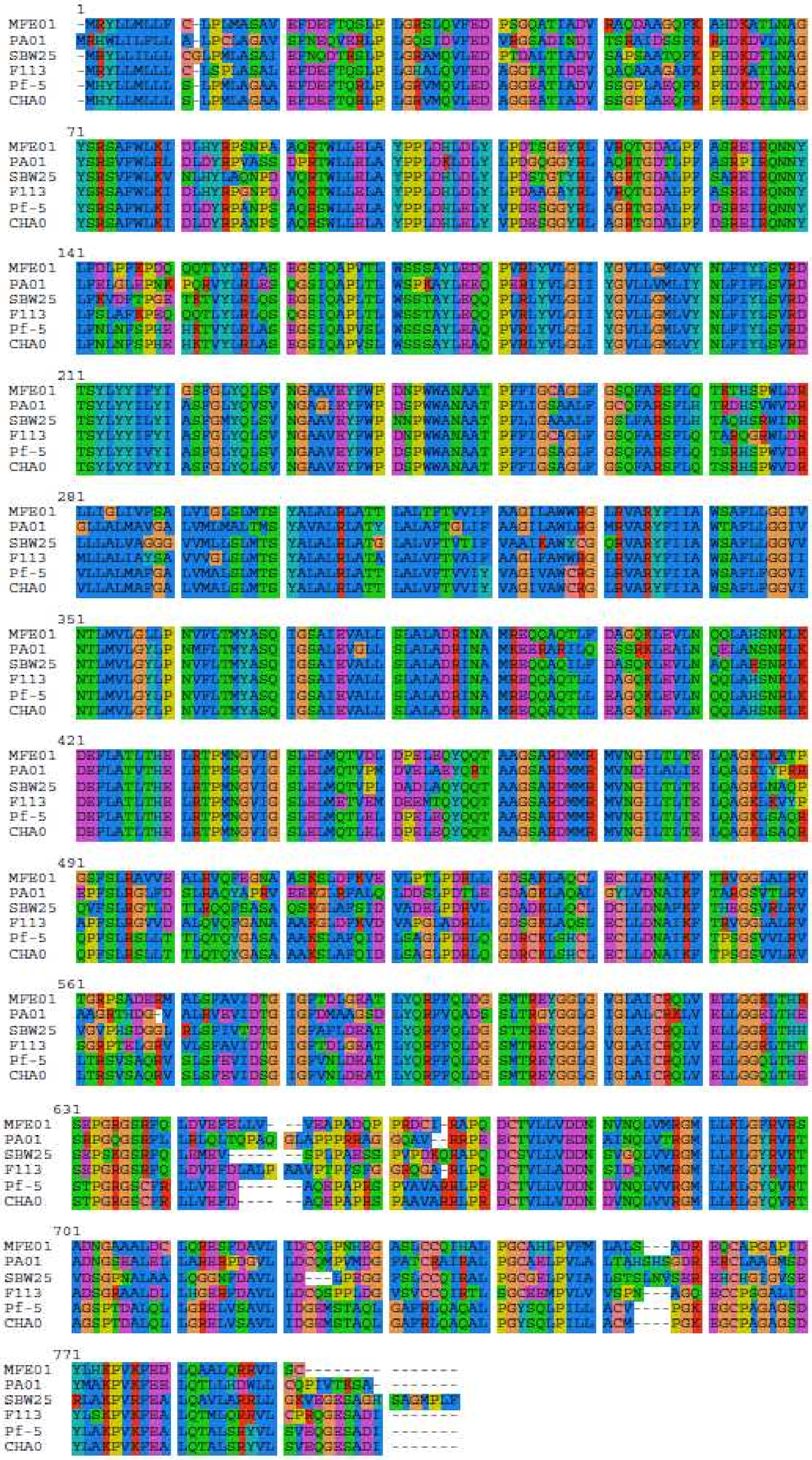

Supplementary Figure 5: Alignment of LadS protein sequences from *Pseudomonas fluorescens* MFE01 with those from different *Pseudomonas*. Except for MFE01, the sequences were retrieved from the "*Pseudomonas* genome database." MFE01: *Pseudomonas fluorescens* MFE01. PAO1: *Pseudomonas aeruginosa* PAO1. SBW25: *Pseudomonas fluorescens* SBW25. F113: *Pseudomonas ogarae* F113. Pf-5: *Pseudomonas protegens* Pf-5. CHA0: *Pseudomonas protegens* CHA0

```
                1
CsrA1_MFE01     ----------  MLILTRKVGE SINIGD-DIT ITILGVSGQQ VRIGINAPKN VAVHREEIYQ RIQAGLTAPD
CsrA2_MFE01     ----------  MLVLSRVVGE LISIGD-DIT LRVLSVNGSS VRFGVEAPKK VHVHRAEVYD RIKR------
CsrA3_MFE01     ----------  MLILTRRCAE SLIIGDGEIT VTVLGVKGNQ VRIGVNAPKE VAVHREEIYL RIKK-----E
RsmA_PAO1       ----------  MLILTRRVGE TLMVGD-DVT VTVLGVKGNQ VRIGVNAPKE VAVHREEIYQ RIQK-----E
CsrA1_SBW25     MSLKVIKEKS  MLILTRKVGE SINIGD-DIT ITILGVSGQQ VRIGINAPKD VAVHREEIYQ RIQAGLTAPD
CsrA2_SBW25     ----------  MLILTRRCAE SLIIGDGEIT VTVLGVKGNQ VRIGVNAPKE VAVHREEIYL RIKK-----E
CsrA3_SBW25     ----------  MLVLSRAVGE VISIGD-DIA VHILELNGSQ VKFGVEAPNG VHVHRAEVYQ KIRDRLGATT
RsmE_F113       ----------  MLILTRKVGE SINIGD-DIT ITILGVSGQQ VRIGINAPKN VAVHREEIYQ RIQAGLTAPD
RsmI_F113       ----------  MLVLSRAVGE LISIGD-DIS VRVLSVSGGT VRFGVEAPRH VDVHRSEIYD KIQKRKALAT
RsmA_F113       ----------  MLILTRRCAE SLIIGDGEIT VTVLGVKGNQ VRIGVNAPKE VAVHREEIYL RIKK-----E
RsmE_Pf-5       ----------  MLILTRKVGE SINIGD-DIT ITILGVSGQQ VRIGINAPKD VAVHREEIYQ RIQAGLTAPD
RsmA_Pf-5       ----------  MLILTRRCAE SLIIGDGEIT VTVLGVKGNQ VRIGVNAPKE VAVHREEIYL RIKK-----E
CsrA1_CHA0      ----------  MLILTRKVGE SINIGD-DIT ITILGVSGQQ VRIGINAPKD VAVHREEIYQ RIQAGLTAPD
CsrA2_CHA0      ----------  MLILTRRCAE SLIIGDGEIT VTVLGVKGNQ VRIGVNAPKE VAVHREEIYL RIKK-----E

               71
CsrA1_MFE01     KPQTP---
CsrA2_MFE01     KEAAAKAG
CsrA3_MFE01     KDEEPSH-
RsmA_PAO1       KDQEPNH-
CsrA1_SBW25     KNQTP---
CsrA2_SBW25     KDEEPSL-
CsrA3_SBW25     HPVPVPNL
RsmE_F113       KPQTP---
RsmI_F113       RKACSVE-
RsmA_F113       KDDEPSH-
RsmE_Pf-5       KRETP---
RsmA_Pf-5       KDEEPSH-
CsrA1_CHA0      KRETP---
CsrA2_CHA0      KDEEPSH-
```

Supplementary Figure 6: Alignment of CrsA-like protein sequences from *Pseudomonas fluorescens* MFE01 with those from different *Pseudomonas*. Except for MFE01, the sequences were retrieved from the "*Pseudomonas* genome database." MFE01: *Pseudomonas fluorescens* MFE01. PAO1: *Pseudomonas aeruginosa* PAO1. SBW25: *Pseudomonas fluorescens* SBW25. F113: *Pseudomonas ogarae* F113. Pf-5: *Pseudomonas protegens* Pf-5. CHA0: *Pseudomonas protegens* CHA0

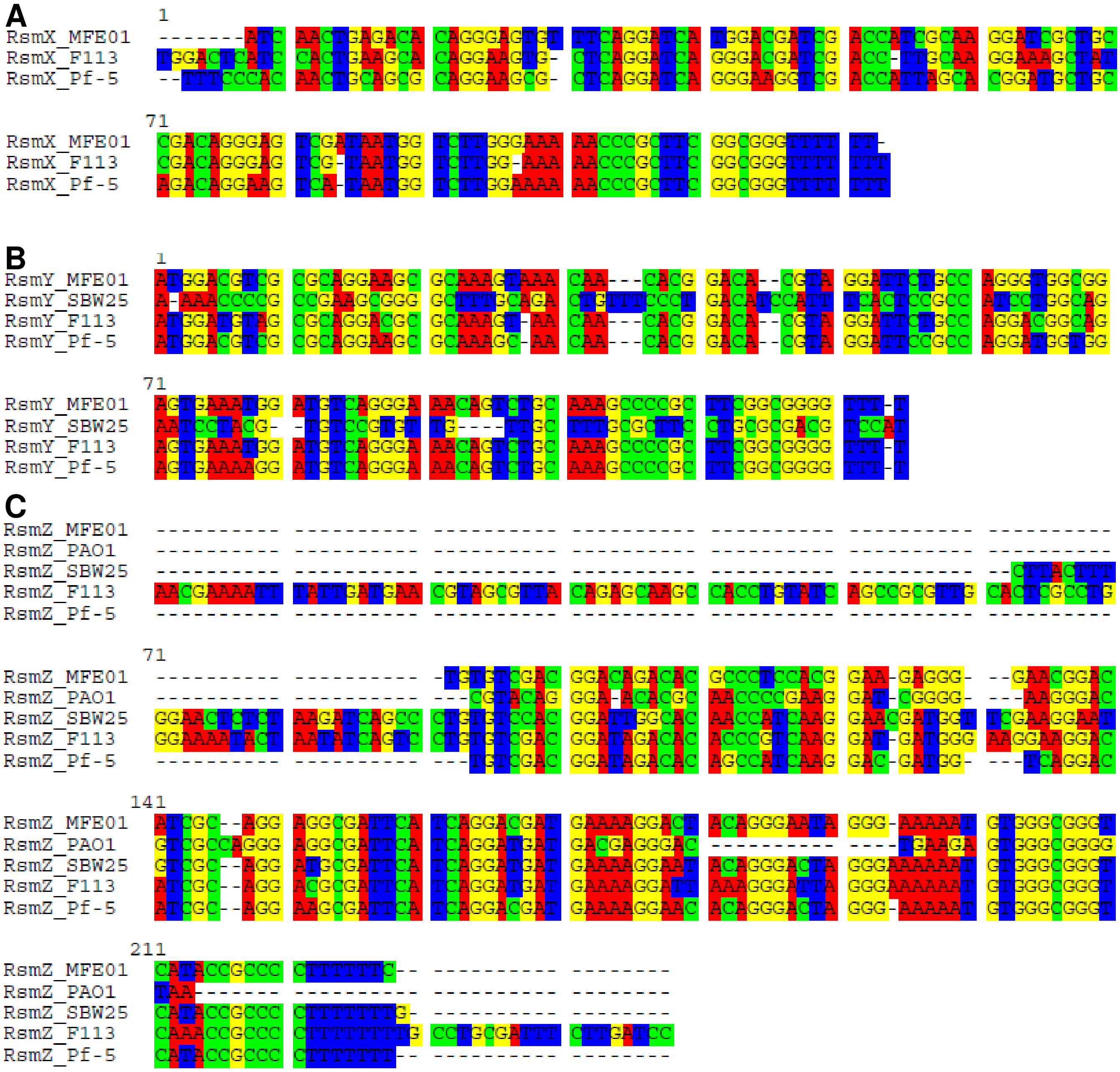

Supplementary Figure 7: Alignments of RsmX, RsmY, and RsmZ ncRNA gene sequences from *Pseudomonas fluorescens* MFE01 with those from different *Pseudomonas* strains.

A. RsmX. **B.** RsmY. **C.** RsmZ. Except for MFE01, the sequences were retrieved from the "*Pseudomonas* genome database." MFE01: *Pseudomonas fluorescens* MFE01. PAO1: *Pseudomonas aeruginosa* PAO1. SBW25: *Pseudomonas fluorescens* SBW25. F113: *Pseudomonas ogarae* F113. Pf-5: *Pseudomonas protegens* Pf-5. CHA0: *Pseudomonas protegens* CHA0

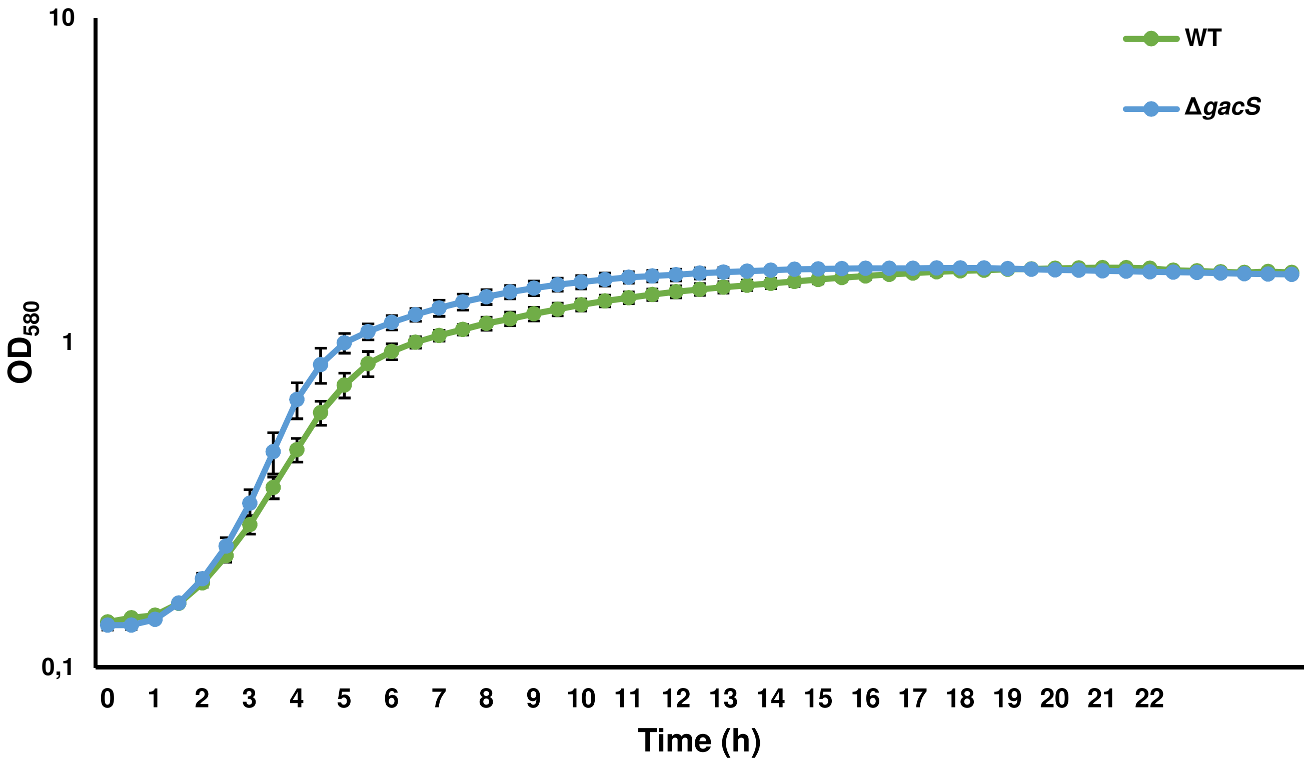

Supplementary Figure 8: Growth curves of MFE01 and its Δ*gacS* mutant.

Growth were performed in LB medium in a 24 wells microplate. Growth were started at OD_{580} = 0,1 in LB medium, 28° C, 180 rpm. Green curve corresponds to the wild-type MFE01 strain and blue curve corresponds to the Δ*gacS* strain. Error bars correspond to standard error (SD). Data obtained from three biological replicates (n=3).

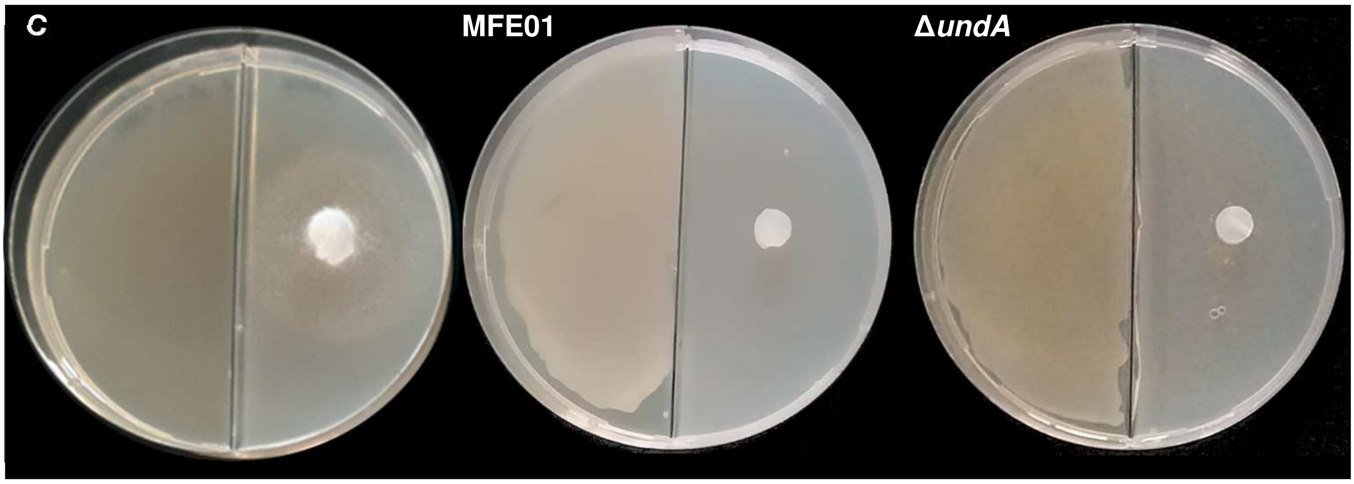

Supplementary Figure 9: *Pseudomonas fluorescens* WT and Δ*undA* strains completely inhibit *Phytophthora infestans*.

A typical photograph depicting an inhibition test of *P. infestans* by VOCs emitted from wild-type MFE01 and its Δ*undA* deletion mutant. The anti-*phytophthora* activity of wild-type MFE01 (MFE01) or the MFE01 Δ*undA* deletion mutant (Δ*undA*), affected only in its emission of 1-undecene, is evaluated in bi-compartmentalized petri-dishes. The growth of *P. infestans* (right compartment) is measured after 7 days of incubation in the presence of volatile molecules emitted by MFE01 or Δ*undA* (left compartment) at room temperature (21° C). The negative control (C) corresponds to an experimental condition where *P. infestans* develops in the absence of volatile molecules emitted by bacteria. Each experiment was repeated 5 times.

tssA operon : cuaucaauuggaugagucgcugaacugcucuucgauacugucccgcguugcugaaacuugauucgcgcgcaugacuguaauccuuaau**aaaggc**cauggaug

undA operon : guguaaugccgcgccuugccuugaacgagaaccccaccuagucucgucauagcgaauucgcccggaaggcgagcgggcagugcucaugca**aggacu**cucaug

hcnABC operon : gcgucgacggugcaaauagagcaagaaaagcgucgacgaggcguagugcagcaaggagcugacacaaccucaacugaaucuucacgg**acgaac**accguaaug

Supplementary Figure 10: Putative RsmA-like proteins binding sites on *tssA*, *undA* and *hcnABC* putative operon mRNAs.

Shine dalgarno sequences (in bold) were predicted by the De Novo DNA RBS calculator (https://www.denovodna.com/software/design_rbs_calculator). Start codon for the first gene of each operon is shown in bleu. The RsmA binding sites described by Chihara et al. 2021, i.e. (G/C/A)N(G/C)(G/C/A)N(C/G/A)(A/C)(U/A/G)GG(A/U/C)(C/A)(G/A/U)N(C/G), were searched, with a maximum of 2 errors, in the 100 bp preceding the start codon of each operon on putative mRNAs and are shown in red. The RsmA binding sequences described by (Duss *et al.* 2014), i.e ANGGA, were search without error and are underlined.
